# Efficient Production of Gene-Modified Mice using *Staphylococcus aureus* Cas9

**DOI:** 10.1038/srep32565

**Published:** 2016-09-02

**Authors:** Xiya Zhang, Puping Liang, Chenhui Ding, Zhen Zhang, Jianwen Zhou, Xiaowei Xie, Rui Huang, Ying Sun, Hongwei Sun, Jinran Zhang, Yanwen Xu, Zhou Songyang, Junjiu Huang

**Affiliations:** 1 State Key Laboratory of Ophthalmology, Zhongshan Ophthalmic Center, Sun Yat-sen University, Guangzhou, 510275, China; 2 Key Laboratory of Gene Engineering of the Ministry of Education, Guangzhou Key Laboratory of Healthy Aging Research and State Key Laboratory of Biocontrol, SYSU-BCM Joint Research Center, School of Life Sciences, Sun Yat-sen University, Guangzhou, 510275, China; 3 Key Laboratory of Reproductive Medicine of Guangdong Province, School of Life Sciences and the First Affiliated Hospital, Sun Yat-sen University, Guangzhou, 510275, China; 4 Department of Pathology, First Affiliated Hospital, Sun Yat-sen University, Guangzhou, 510080, China

## Abstract

The CRISPR/Cas system is an efficient genome-editing tool to modify genes in mouse zygotes. However, only the *Streptococcus pyogenes* Cas9 (SpCas9) has been systematically tested for generating gene-modified mice. The protospacer adjacent motif (PAM, 5′-NGG-3′) recognized by SpCas9 limits the number of potential target sites for this system. *Staphylococcus aureus* Cas9 (SaCas9), with its smaller size and unique PAM (5′-NNGRRT-3′) preferences, presents an alternative for genome editing in zygotes. Here, we showed that SaCas9 could efficiently and specifically edit the X-linked gene *Slx2* and the autosomal gene *Zp1* in mouse zygotes. SaCas9-mediated disruption of the tyrosinase (*Tyr*) gene led to C57BL/6J mice with mosaic coat color. Furthermore, multiplex targeting proved efficient multiple genes disruption when we co-injected gRNAs targeting *Slx2*, *Zp1*, and *Tyr* together with SaCas9 mRNA. We were also able to insert a Flag tag at the C-terminus of histone *H1c*, when a Flag-encoding single-stranded DNA oligo was co-introduced into mouse zygotes with SaCas9 mRNA and the gRNA. These results indicate that SaCas9 can specifically cleave the target gene locus, leading to successful gene knock-out and precise knock-in in mouse zygotes, and highlight the potential of using SaCas9 for genome editing in preimplantation embryos and producing gene-modified animal models.

Gene-modified mouse models are important tools in understanding gene function *in vivo* and developing new therapeutics for diseases. Before the advent of nuclease-based genome-editing tools, gene-modified mice were generated mainly by gene targeting in embryonic stem cells (ESCs), a time-consuming and labour-intensive process^1^. Several classes of artificial nucleases have been developed, including zinc-finger nucleases (ZFN), transcription activator-like effector nucleases (TALEN), and the CRISPR/Cas system^2^,^3^. These nucleases have been shown to work efficiently in zygotes from different species, and not restricted to ESCs, thus enabling one-step generation of gene-modified animals with drastically improved efficiency and reduced cost and time commitment^4^,^5^,^6^,^7^,^8^,^9^,^10^,^11^,^12^. In zygotes, the nucleases induce double-strand breaks (DSBs) at target sites, which are subsequently repaired through either non-homologous end joining (NHEJ) or homology-directed repair (HDR), resulting in possible frame-shift mutations and precise gene modifications, respectively^2^,^13^. ZFN and TALEN are bipartite fusion proteins consisting of nuclease domains as well as DNA-binding domains that recognize the target site by protein-DNA interaction^2^. Complex protein engineering steps are required to generate site-specific ZFN and TALEN, which has greatly hampered the application and expansion of these platforms^3^.

The CRISPR/Cas system, which consists of the Cas nuclease and guide RNA (gRNA), is adapted from the acquired immune systems of bacteria and archaea^14^,^15^,^16^. The 20-nucleotide sequence at the 5′ or 3′ end of the gRNA specifies the target site through RNA-DNA hybridization^17^,^18^,^19^. As a result, targeting different genes using CRISPR/Cas can be more easily achieved through the manipulation of gRNA sequences. Its simplicity, flexibility, and high efficiency has enabled the application of CRISPR/Cas systems in generating a variety of gene-modified species within a short amount of time^4^,^6^,^10^,^11^,^17^,^18^,^19^,^20^,^21^,^22^,^23^,^24^. Of the different CRISPR/Cas systems, such as *Streptococcus pyogenes* Cas9 (SpCas9), *Staphylococcus aureus* Cas9 (SaCas9), *Lachnospiraceae* Cpf1 (LbCpf1), *Acidaminococcus* Cpf1 (AsCpf1), *Streptococcus thermophilus* Cas9 (StCas9), and *Neisseria meningitidis* Cas9 (NmCas9), only SpCas9 has been extensively characterized and widely used in animal zygotes genome editing^6^,^7^,^20^,^21^,^22^,^24^,^25^,^26^,^27^. However, its specific protospacer adjacent motif (PAM, 5′-NGG-3′) preference limits the number of potential genome recognition sites for SpCas9, which calls for alternative CRISPR/Cas systems for genome editing in zygotes.

SaCas9 is derived from *Staphylococcus aureus*, pathogenic bacteria that can cause human skin and soft tissue infections^28^,^29^,^30^. SaCas9 is the smallest among all the known Cas nucleases, largely due to its small recognition (REC) lobe^28^. This small size makes it particular attractive for gene therapy, which often involves *in vivo* delivery using viral vectors. In addition, SaCas9 recognizes a unique 5′-NNGRRT-3′ PAM sequence through its PAM-interacting (PI) domain^29^. Moreover, the PAM sequence of SaCas9 is predicted to occur once every 32 bps, compared to once every 8 bps for SpCas9. These unique properties suggest specific recognition and cleavage by SaCas9, which is supported by the genome-wide sequencing data reported recently^31^. Despite its promise in genome editing, no reports to date have examined the ability of SaCas9 to modify genomes in zygotes.

In this report, we compared the genome editing efficiency and specificity of SaCas9 vs. SpCas9 in mouse zygotes, and examined the resultant mosaicism. In addition, we also provided evidence that demonstrated the feasibility of using SaCas9 for multiplex gene disruption and homology-directed repair in mouse embryos. Our data reveal that SaCas9 is comparable to SpCas9 in genome editing in animal zygotes, and highlight the potential of SaCas9 as an alternative to SpCas9 for modifying genomes.

## Results

### SaCas9 cleaves target sites efficiently in mouse embryos

**Figure 1:**
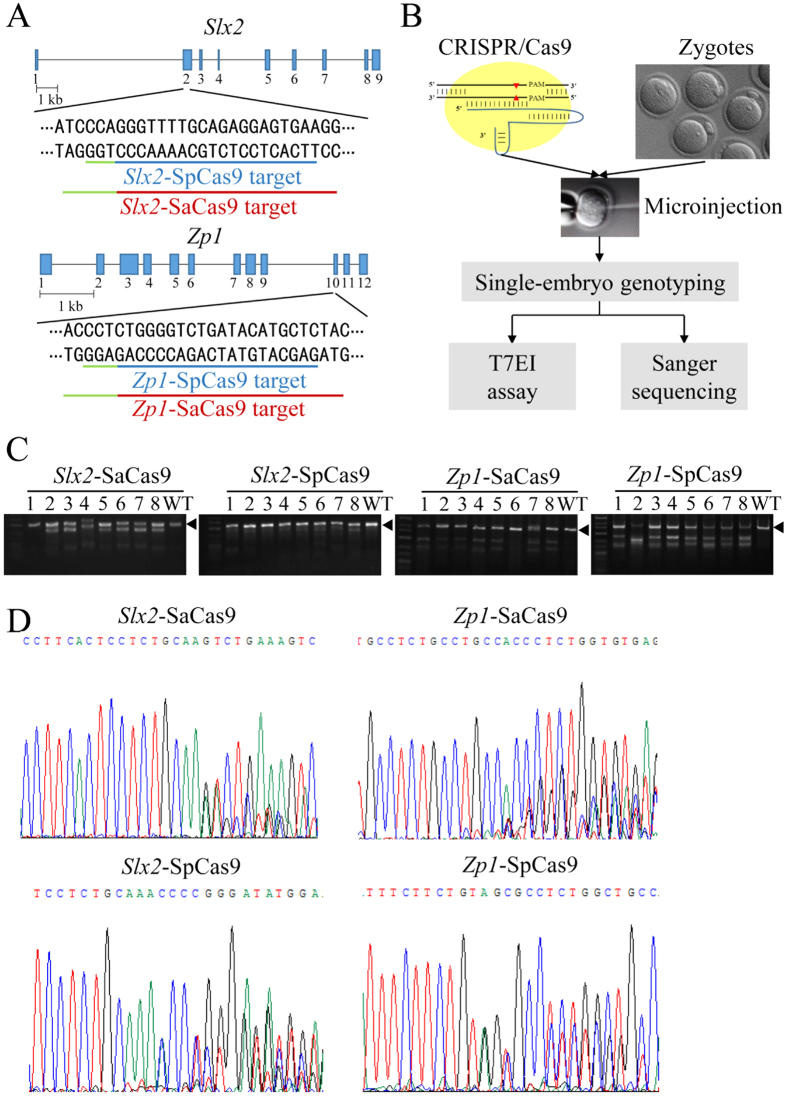
SaCas9 can efficiently cleave target genes in mouse embryos. (**A**) Schematic representations of the *Slx2* and *Zp1* target sites. Here, SaCas9 and SpCas9 target the same regions of *Slx2* and *Zp1*, their target sequences are underlined respectively in red and blue. PAM sequences are underlined in green. (**B**) A flow chart indicating the strategy to determine the cleavage efficiencies of SaCas9. SpCas9 was used for comparsion. Cas9 mRNAs and their respective gRNAs were injected into 0.5-day old zygotes. After 48 hours, a portion of the injected embryos were *in vitro* cultured for the T7 endonuclease I (T7EI) assay and Sanger sequencing. (**C**) The regions spanning the target sites of the *Slx2* and *Zp1* loci were PCR amplified. The PCR products were then used for the T7EI assay. Black arrowheads indicate PCR fragments that contain no mismatch. WT, wild-type. (**D**) The PCR products from (**C**) were also examined by Sanger sequencing. Representative sequencing results of SaCas9 or SpCas9-cleaved embryos are presented here. The presences of multiple peaks near the PAM sequences indicate sites of cleavage and NHEJ-mediated repair.

To test whether SaCas9 could edit genes in mouse zygotes, we designed two *Staphylococcus aureus* gRNAs (Sa-gRNAs) to respectively target the X-linked gene *Slx2* and autosomal gene *Zp1* (Fig. 1A). For comparison, *Streptococcus pyogenes* gRNAs (Sp-gRNAs) which could also target *Slx2* and *Zp1* were designed as well (Fig. 1A). Next, we injected the gRNAs with the mRNA of their respective Cas9 nucleases into 0.5-day mouse zygotes. The injected embryos were either transferred into 0.5-day pseudopregnant mice 2 hours after microinjection or cultured *in vitro* and genotyped. The cultured embryos were harvested 48 hours later to PCR amplify the target regions for the T7 endonuclease I (T7EI) assay and Sanger sequencing (Fig. 1B). As shown in Fig. 1C, the T7EI assay indicated that SaCas9 was able to cleave both the *Slx2* and *Zp1* loci efficiently, at levels comparable to SpCas9. Sanger sequencing of the PCR amplicons further revealed that 24/27 (88.8%) of the embryos had been cleaved by *Slx2*-SaCas9, compared to 14/26 (53.8%) for *Slx2*-SpCas9, and 23/25 (92%) of the embryos cleaved by *Zp1*-SaCas9 vs. 26/27 (96.3%) by *Zp1*-SpCas9 (Fig. 1D and Table 1). These results demonstrate the feasibility of using SaCas9 to edit target genes in mouse zygotes.

### Efficient generation of mutant mice by SaCas9

**Figure 2:**
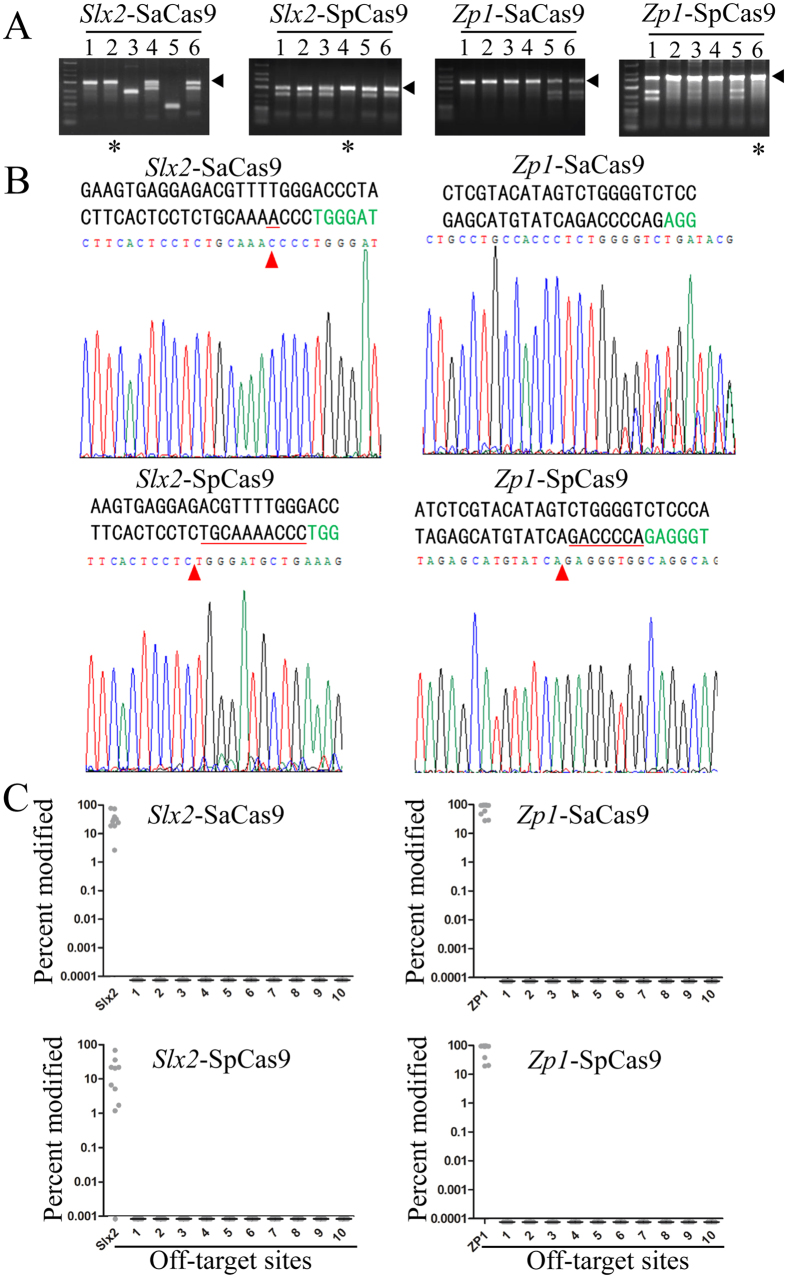
SaCas9 can efficiently generate mutant mice. The injected embryos from Fig. 1 were transplanted into pseudopregnant mice to generate mutant mice. (**A**) PCR amplification and T7EI assays were carried out as described above to identify mutant founder mice. Arrowheads denote uncut PCR products with no mismatch. *Indicates pups that appeared to be wild-type in the T7EI assay, but turned out to be homozygous mutants when analysed by Sanger sequencing. (**B**) Representative sequencing chromatographs are presented here, with the target sequences (PAM in green) shown above. Most mutant mice resemble pup #5 from the *Zp1*-SaCas9 group, where multiple peaks are present near the PAM sequence. Others resemble pup #2 from the *Slx2*-SaCas9 group, #4 from *Slx2*-SpCas9, and #6 from *Zp1*-SpCas9, in which deletions of various sizes (1–10 bps) were apparent. These are the same samples that were deemed uncut (wild-type) in the T7EI assay in (**A**). Red arrowheads indicate sites with base pair deletion, with the deleted residues underlined in red. (**C**) Ten pups were randomly selected from each group. Regions encompassing the top ten predicted off-target sites (numbered on the x-axis) were PCR amplified using the genomic DNA from these pups and analysed by deep sequencing. The percentages of modifications at each site, including the intended target, are plotted on the y-axis. Only 9 samples from *Slx2*-SaCas9 were plotted here because of insufficient sequencing depth in one sample. Of the *Slx2*-SpCas9 group, one supposedly “wild-type” pup was actually cleaved by SpCas9 at a very low frequency, while the other one was not cleaved at all. For both *Slx2*-SaCas9 and *Slx2*-SpCas9, the cleavage efficiency appeared low in several samples.

Following our *in vitro* analysis of SaCas9-mediated genome editing in embryos, we went on to evaluate the efficiency and specificity of SaCas9 in mutant mice. The birth rates of mice 20 days after the transplantation of SaCas9 and SpCas9 modified embryos were calculated and shown in Table 1, where comparable birth rates of SaCas9 vs. SpCas9 were found (17.9% and 8.3% compared to 9.5% and 16.5%). This result indicates low embryo toxicity for SaCas9. Preliminary T7EI analysis of the pups indicated the presence of mutant mice as a result of cleavage by SaCas9 or SpCas9 (Fig. 2A). Further verification by Sanger sequencing showed that some of pups that had been deemed wild-type by the T7EI assay were actually homozygous mutant mice (Fig. 2B). As a result, all pups were verified by Sanger sequencing to more accurately estimate Cas9 efficiency. Again, SaCas9 appeared comparable to SpCas9, 16/17 (94.1%) pups were edited by SaCas9 as opposed to 8/17 (47.1%) by SpCas9 for the *Slx2* locus, and 14/18 (77.7%) vs. 10/12 (83.3%) for the *Zp1* locus (Table 1). These results suggest that as has been found for SpCas9, the efficiency of SaCas9 is site dependent and that the overall efficiency of SaCas9 is similar to that of SpCas9^6^.

The unique 5′-NNGRRT-3′ PAM preference of SaCas9 should in theory lead to fewer off-target sites than SpCas9. To further compare the specificity of SaCas9 and SpCas9 in founder mice, we selected 10 mutant pups from each group, except that eight mutant and two wild-type (WT) pups (verified by Sanger sequencing) (Table 1) were chosen for the *Slx2*-SpCas9 group. On-target sites of *Slx2* and *Zp1* as well as the top ten potential off-target sites were PCR amplified and deep sequenced (Fig. 2C). None of the potential off-target sites examined appeared to be cleaved by either SaCas9 or SpCas9, suggesting that both Cas9 nucleases are very specific. It is interesting to note that deep sequencing revealed very low gene modification efficiency (1.71%) in one pup, a “wild-type” mouse in the *Slx2*-SpCas9 group as verified by Sanger sequencing, likely because deep sequencing is much more sensitive than Sanger sequencing at detecting indels.

### Mutant founder mice generated by SaCas9 are mosaic

**Figure 3:**
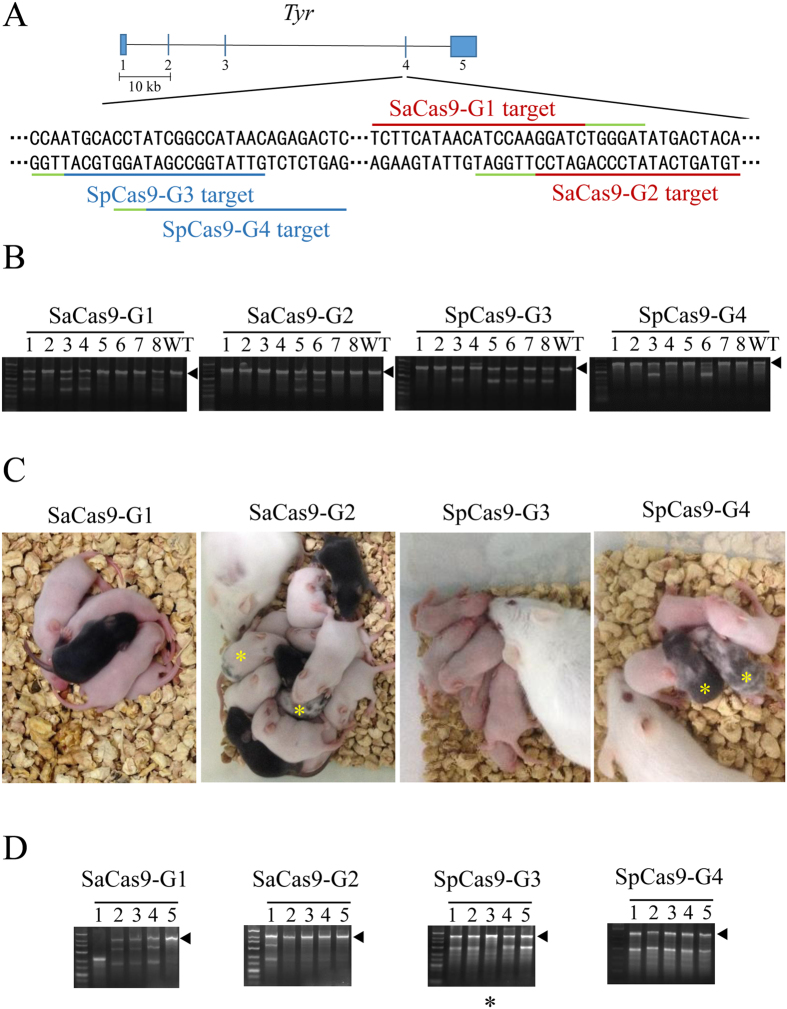
Founder mice generated using SaCas9 are mosaic. (**A**) A schematic representation of the target sites in the tyrosinase (*Tyr*) gene. The SaCas9 and SpCas9 target sequences are respectively underlined in red and blue, with PAM sequences underlined in green. (**B**) T7EI analysis of the injected embryos was performed to determine the cleavage efficiency of SaCas9 and SpCas9 at the *Tyr* locus. Arrowheads indicate uncut (or no mismatch) PCR products. WT, wild-type. (**C**) Coat colour was assessed in founder mice nine days after birth. Albino and mosaic (yellow*) founders were apparent in mice obtained from embyros modified by SaCas9 or SpCas9. (**D**) Genomic DNA was extracted from newborn mice and used in T7EI assays to assess Cas9 cleavage at the *Tyr* locus. Arrowheads indicate uncut (or no mismatch) PCR products. *Indicates pups that appeared to be wild-type in the T7EI assay, but turned out to be homozygous mutants when analysed by Sanger sequencing.

One limitation of using SpCas9 in generating gene-modified mice is that most of the founder mice are mosaic, which complicates phenotypic analysis of founder mice. To determine whether SaCas9 had similar issues, we designed 2 Sa-gRNAs (SaCas9-G1 and SaCas9-G2) and 2 Sp-gRNAs (SpCas9-G3 and SpCas9-G4) to target exon 4 of the tyrosinase gene (*Tyr*) (Fig. 3A). Tyrosinase is a key enzyme for melanin production whose dysfunction leads to albinism in C57BL/6J mice. Albinism in C57BL/6J mice makes it easy to visualize mosaicism in the offspring^32^. Most of the injected zygotes were transferred into pseudopregnant mothers. We genotyped 8 embryos using the T7EI assay after 48 hours of *in vitro* culture and found that both SaCas9 and SpCas9 could edit the *Tyr* gene (Fig. 3B). About 9 days after birth, both mosaic coloured and totally albino pups could be found in SaCas9 and SpCas9 groups (Fig. 3C). These albino and mosaic pups were confirmed to be C57 by microsatellite analysis (Supplementary information, Fig. S1). For SpCas9, the percentages of albino pups were 66.7% and 68.8% (Table 2). For SaCas9, the albino rate was 72.7% for SaCas9-G1, but 38.1% for SaCas9-G2. These data indicate that SaCas9-generated founder mice are mosaic, in agreement with the sequencing results of *Tyr* loci in the pups (Supplementary information, Fig. S2).

To further compare the efficiency of SaCas9 and SpCas9-mediated mutant mice generation, the genotypes of these pups were examined by both T7EI assay and Sanger sequencing (Fig. 3D, and Supplementary information, Fig. S3), which found that 3 black pups had actually been cleaved by SaCas9 at the *Tyr* locus (Table 2). The efficiency of SaCas9-G1 and SaCas9-G2 were 81.8% and 61.9%, respectively, comparable to that of SpCas9-G3 (66.7%) and SpCas9-G4 (81.3%) (Table 2). Again, these data underline the site dependence of the genome editing efficiency of SaCas9.

### Multiplex gene disruption in mouse embryos by SaCas9

**Figure 4:**
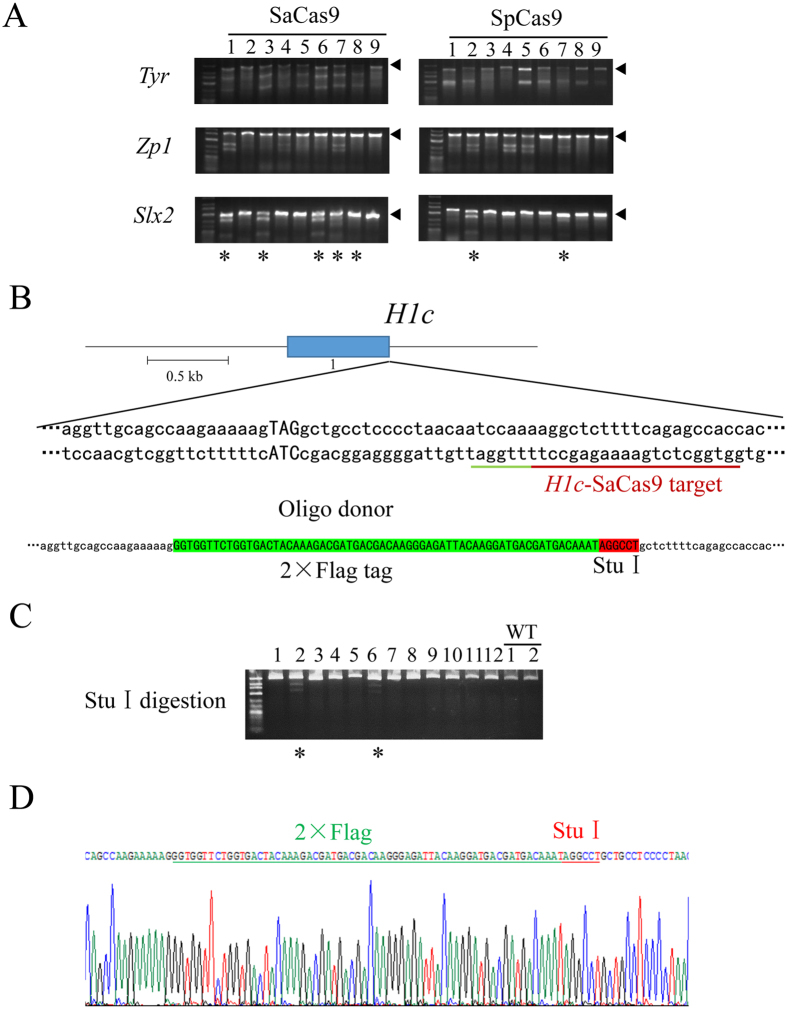
SaCas9-mediated multiplex gene disruption and homology directed repair (HDR) in mouse embryos. (**A**) gRNAs targeting *Tyr*, *Zp1*, and *Slx2* were simultaneously injected into mouse zygotes with their respective Cas9 mRNAs. Regions spanning the target sites were PCR amplified for T7EI analysis. Arrowheads indicate uncut (or no mismatch) PCR products. All embryos were further genotyped by Sanger sequencing. *Indicates triple targeted embryos. Sanger sequencing revealed that #2, #3, #6 and #8 embryos from *Zp1* and #8 embryo from *Slx2* (both in the SaCas9 group) had homozygous deletions. And Tyr and Zp1 from #8 embryos of SpCas9 group also had homozygous deletions. (**B**) The design of the gRNA and single-stranded DNA oligo for the *H1c* locus. The target site is underlined in red, and the PAM sequence in green. The 2 × Flag tag coding sequence is in green and StuI site in red. (**C**) The region encompassing the target site of the *H1c* locus was PCR amplified and then digested with StuI for RFLP (Restriction fragment length polymorphism) analysis. *Indicates embryos repaired by HDR. (**D**) PCR products from (**C**) were TA cloned for sequence verification of successful knock-in. A Representative sequencing chromatograph is shown. The 2 × Flag tag sequence and StuI site are underlined respectively in green and red.

Our data thus far indicated that SaCas9 can cleave efficiently in a single gene locus in mouse embryos. We next addressed whether SaCas9 could disrupt multiple genes simultaneously. SaCas9 mRNA and multiple gRNAs that target different loci (*Slx2*-SaCas9, *Zp1*-SaCas9, and *Tyr*-SaCas9-G1) were co-injected into the cytoplasm of mouse zygotes. SpCas9 mRNA and the corresponding SpCas9 gRNAs (*Slx2*-SpCas9, *Zp1*-SpCas9 and *Tyr*-SpCas9-G3) were also injected into zygotes as a control. The injected embryos were harvested 48 hours later for whole-genome amplification by multiplex displacement amplification (MDA) and the target regions were PCR amplified and genotyped (Fig. 4A and Supplementary information, Fig. S4). Five embryos from the SaCas9 group (5/9, 55.6%) had been targeted at all three loci. In contrast, only 2/9 (22.2%) embryos from the SpCas9 group were triple targeted (Fig. 4A). The genotypes, including single- and double-targeted embryos, are summarized in Table 3. Our data revealed that 46.7% (14/30) of SaCas9-injected embryos were triple targeted, while only 8.1% (3/37) of SpCas9-injected embryos were triple-targeted (Table 3 and Supplementary information, Table S1). These results demonstrate the feasibility of multiplex gene disruption using SaCas9.

### Precise gene knock-in modification in mouse embryos mediated by SaCas9

Our results clearly show that SaCas9 can generate indels via the NHEJ pathway, which leads to gene knockout (KO). Next, we explored the possibility of using SaCas9 to generate knock-in (KI) mice, which requires a donor template and utilizes the HDR mechanism. We designed a Sa-gRNA to target the 3′ end of the histone *H1c* coding sequence, and a single-stranded DNA oligo encoding a 2 × Flag tag and a StuI restriction site flanked by homology arms (Fig. 4B). We co-injected the DNA oligo and gRNA together with SaCas9 mRNA into zygotes and collected the embryos after 48 hours to PCR amplify the *H1c* target region. If a PCR product could be digested with StuI, it would indicate successful insertion of the oligo sequence. Indeed, 2 out of 12 embryos appeared to have incorporated the StuI site (Fig. 4C). The PCR products were subsequently TA cloned for Sanger sequencing (Fig. 4D). We found that sequences for the 2 × Flag tag as well as the StuI site were both integrated into the *H1c* gene locus, supporting the notion that SaCas9 can efficiently mediate gene knock-in through the HDR mechanism.

## Discussion

The rapid development and application of CRISPR/Cas9 technology has led to numerous studies on its efficacy and specificity, although the majority of studies have focused on SpCas9. Here, we describe the first attempt to systematically determine the genome editing efficiency and specificity of SaCas9 in mouse embryos. Using multiple assays and platforms, we showed that the efficiency of SaCas9 was comparable to that of SpCas9. This finding is in agreement with results reported for human cell lines^33^. Three independent studies have found SaCas9 to be specific at different target sites in human cells^29^,^31^,^33^. Our deep sequencing results also support high specificity for SaCas9 in mouse embryos. Multiplex gene disruption and one-step knock-in are some of the key advantages of SpCas9. By simultaneously targeting three genes, we demonstrated that SaCas9 was also capable of multiplex gene disruption. And we successfully tagged endogenous histone *H1c* on the c-terminus with a 2 × Flag tag. It has been well documented, although not fully understood, that Cas9/gRNA specificity often depends on the particular sequences and/or structures of the regions being targeted. Therefore, extensive high-throughput sequencing investigations (e.g., using GUIDE-seq or BLESS) are sorely needed to better elucidate the exact factors that determine the specificity of SaCas9 and SpCas9 at different genomic sites. And the efficiency of SaCas9-mediated multiplex gene disruption and gene knock-in in comparison with SpCas9 warrants further investigation. Mosaicism has been a common phenomenon in SpCas9-generated founder mice, where an animal is a chimera of both wild-type and mutant cells^3^,^34^. It is mainly caused by inefficient cleavage of the target site in one-cell stage embryos and prolonged nuclease activities following the first round of cell division^34^. Whether SaCas9 could also lead to mosaicism had been largely unknown. By targeting the coat-colour gene tyrosinase (*Tyr*), we found that SaCas9-generated founder mice also exhibited mosaicism, similar to those generated by SpCas9^32^.

Our work on SaCas9 in mouse models indicates that SaCas9, similar to SpCas9, can be harnessed to edit genomes of other animals such as rats, pigs, and monkeys^4^,^10^,^20^,^22^,^23^,^24^,^35^. Dr. Yong Fan’s group and ours have shown that SpCas9 could edit CCR5 andβ-globin genes in human tripronuclear zygotes^36^,^37^. The UK Human Fertilisation and Embryology Authority (HFEA) recently licensed Dr. Kathy Niakan to edit human embryo genomes for the mechanistic study of human preimplantation embryos^38^. SaCas9 may be an attractive alternative to SpCas9 in similar studies. Indeed, experiments have been carried out to optimize SaCas9 and expand its applications. The structure and catalytic residues of SaCas9 have been reported, and nuclease-dead SaCas9 has been created^28^. When fused with the KRAB (Krüppel-associated box) domain of KOX1, VP16, the histone acetyltransferase (HAT) core domain of P300, or the DNA methylation domain of DNMT3A, nuclease-dead SaCas9 may enable precise transcriptional and epigenetic regulation^28^,^39^,^40^,^41^,^42^. Dr. J. Keith Joung and colleagues reported an engineered SaCas9 protein that recognized the 5′-NNNRRT-3′ PAM sequence, potentially broadening the targetable sites of SaCas9^31^. Dr. Feng Zhang’s group developed a SaCas9 variant with higher specificity by replacing several positively charged residues with uncharged amino acids^43^. Continued improvements should greatly enhance our ability to use SaCas9 in research, broaden its applications in other fields, and facilitate potential clinical implementations of CRISPR/Cas9.

## Materials and Methods

### Ethics Statement

All experiments were conducted with protocols approved by The Institutional Animal Care and Use Committee at Sun Yat-sen University.

### Animal Experiments

Mice were purchased from Guangdong Medical Laboratory Animal Center. All mice were housed under climate-controlled conditions (22 ± 1 °C) in a specific pathogen-free animal facility with 14-hour light/10-hour dark cycles in Sun Yat-sen University. C57BL/6J mice (6–8 weeks old females) were superovulated and mated with C57BL/6J males. Plugged females were sacrificed by cervical dislocation. 0.5-day zygotes were collected using potassium simplex optimized medium (KSOM) containing *N*-2-Hydroxyethylpiperazine-*N*′-2-ethanesulfonic acid and sodium bicarbonate (HKSOM), and cultured in KSOM until genotyping or transplantation. The embryos were *in vitro* cultured for 48 hours before genotyping or whole genome amplification. CD1 female mice (6–8 weeks old) that were mated with sterilized CD1 male mice were used as foster mothers.

### *In vitro* transcription of Cas9 mRNA and gRNA

The pT7-3 × Flag-hCas9 plasmid was constructed as reported previously^37^. The transcription template of SaCas9 mRNA was PCR amplified from pX601 (Addgene) using the primer listed in Supplementary information, Table S2. We added the T7 promoter sequence to the 5′ end of the SaCas9 forward primer. The transcription templates were then purified using the PCR Clean Up Kit (Qiagen). SpCas9 and SaCas9 were transcribed using the mMESSAGE mMACHINE T7 ULTRA kit (Life Technologies) following the manufacturer’s instruction. Sequences for SpCas9 gRNAs were cloned into the pDR274 vector (Addgene), using primers listed in Supplementary information Table S2. These recombinant vectors were then linearized with Dra I (NEB) and transcribed using the MEGAshortscript T7 kit (Life Technologies) following the manufacturer’s instruction. The transcription templates of SaCas9 gRNAs were PCR amplified from pX601 (Addgene) using primers listed in Supplementary information, Table S2. We also added the T7 promoter sequence to the 5′ end of the gRNA forward primer. The gRNAs were then transcribed using the MEGAshortscript T7 kit (Life Technologies). Cas9 mRNAs and the gRNAs were subsequently purified using the MEGAclear kit (Life Technologies) and resuspended in RNase-free water.

### Intracytoplasmic injection of Cas9 mRNA and gRNA

The mixture of Cas9 mRNA (200 ng/μl) and gRNA (100 ng/μl) was injected into 0.5-day zygotes of C57BL/6J mice. About 2 hours after injection, the injected zygotes were transplanted into the oviduct of 0.5-day pseudopregnant mothers. Primers used for *in vitro* transcription of Cas9 mRNA and gRNA are listed in Supplementary information, Table S2.

### Single embryo PCR amplification and mouse genotyping

Single embryo PCR amplification was performed as described by Ran *et al*.^44^. Briefly, each embryo was transferred into a PCR tube containing 1 μl lysis buffer, and then incubated at 65 °C for 3 hours followed by 95 °C for 10 min. The lysis product was then amplified using primers listed in Supplementary information, Table S3. Mouse genotyping was done by PCR and sequencing of tail-snips using the Mouse Genotyping Kit (KAPA Biosystems) following the manufacturer’s instruction. Primers used for genotyping are listed in Supplementary information, Table S3.

### Genomic DNA analysis

Target sites were PCR amplified using primers listed in Supplementary information, Table S3. The PCR products were then used in the T7 endonuclease I (T7EI) cleavage assay as described by Shen *et al*.^45^. Primers for direct sequencing of the PCR products, which reveals the presence of double peaks or indels, are listed in Supplementary information, Table S3. PCR products with double peaks were then TA cloned into the pGEM-T vector (Promega) for plasmid DNA extraction and Sanger sequencing.

### Microsatellite analysis

The D7Mit145 site and D12Mit136 site were amplified from albino and mosaic founders. The PCR products were then resolved by electrophoresis on a 10% PAGE gel. Information of primers were in Supplementary information, Table S4.

### Whole genome amplification of embryonic genome DNA

Whole genome amplification of the embryos was performed using the PEPLI-g Midi Kit (Qiagen). Briefly, embryos were transferred into PCR tubes containing reconstituted buffer D2 (7 μl), and then incubated at 65 °C for 10 min. Then STOP solution (3.5 μl) was added to the reaction mixture, followed by addition of the MDA master mix (40 μl) and incubation at 30 °C for 8 hours. The reaction product was diluted with ddH_2_O (3:100), and 1 μl of the diluted DNA was used for PCR amplification. PCR products were then analyzed by the T7EI assay and Sanger sequencing.

### Analysis of off-target sites by deep sequencing

We used the online tool Cas-OFFinder (http://www.rgenome.net/cas-offinder/) to identify potential off-target sites. The mismatch number and bulge size values are: Slx2-SpCas9, 4 and 0 respectively; Slx2-SaCas9, 5 and 0 respectively; *Zp1*-SpCas9, 2 and 0 respectively; and *Zp1*-SaCas9, 6 and 0 respectively. Sequences surrounding these sites were PCR amplified and deep sequenced using Illumina Hiseq 2500 PE250 as paired-end 250 reads. The primers for off-target effect analysis are listed in Supplementary information, Table S5. High-throughput sequencing data were analyzed as previously reported^46^. Briefly, sequencing reads were mapped to the mouse reference genome (UCSC, mm10) by means of BWA with default parameters (v0.7.8). Samtools and Picard tools were used to build index and sort mapped reads by GATK (Genome Analysis ToolKit, version 3.1–1). HaplotypeCaller was used to call variants and each then was divided into indels and SNVs in virtue of SelectVariants. We then excluded indels in the flanking region (±20bp) that did not overlap with the predicted on- and off-target sites by Bedtools. Indel site and indel frequency were calculated. We included 1-bp indel mutations only if they occurred directly adjacent to the cleavage site as reported^46^.

## Additional Information

**How to cite this article**: Zhang, X. *et al*. Efficient Production of Gene-Modified Mice using *Staphylococcus aureus* Cas9. *Sci. Rep*. **6**, 32565; doi: 10.1038/srep32565 (2016).

## Supplementary Material

Supplementary Information

## Figures and Tables

**Table 1:** SaCas9 and SpCas9-mediated mutant mice generation.

Target gene	Cas9	Survived/Injected (%)	Survived embryos		Pups (birth rate)	Mutant pups (%)
			Embryo Genotyped#	Transferred		
*Slx2*	SaCas9	122/166^a^ (73.5%)	24/27	95	17 (17.9%)	16/17 (94.1%)
	SpCas9	204/280^b^ (72.9%)	14/26	178	17 (9.5%)	8/17 (47.1%)
*Zp1*	SaCas9	241/266^a^ (90.6%)	23/25	216	18 (8.3%)	14/18 (77.7%)
	SpCas9	130/136^b^ (95.6%)	26/27	103	17* (16.5%)	10/12 (83.3%)

Note: All the pups were genotyped by Sanger sequencing.

^#^Mutant embryos/embryos tested.

^*^5 newborns were cannibalized by the mother mouse. a *vs* b, p > 0.05, Statistical significance was tested using χ^2^ test.

**Table 2:** Generation of *Tyr* mutant founder mice by SaCas9 and SpCas9.

Cas9	gRNA	Pups/Transferred (%)	Albino pups (%)	Coat color Mosaic pups (%)	Mutant black pups/Black pups	Mutant pups (%)
SaCas9	G1	11/75 (14.7%)	8 (72.7%)	0 (0%)	1/3	9 (81.8%)
	G2	21/158 (13.3%)	8 (38.1%)	3 (14.3%)	2/10	13 (61.9%)
SpCas9	G3	12/99 (12.1%)	8 (66.7%)	0 (0%)	0/4	8 (66.7%)
	G4	16/123 (13.0%)	11 (68.8%)	2 (12.5%)	0/3	13 (81.3%)

**Table 3:** Multiplex gene disruption in mouse embryos by SaCas9 and SpCas9.

Cas9	gRNA	Total embryo No.	Triple-Targeted (%)	Double-Targeted (%)	Single-Targeted (%)	Non-Targeted (%)
SaCas9	*Tyr*-G1	30	14 (46.7%)	11 (36.7%)	3 (10.0%)	2 (6.7%)
	*Zp1*					
	*Slx2*					
SpCas9	*Tyr*-G3	37	3 (8.1%)	11 (29.7%)	17 (50.0%)	6 (16.2%)
	*Zp1*					
	*Slx2*					
