# Supplementary material for: Efficient Production of Gene-Modified Mice using Staphylococcus aureus Cas9
Source: Sci Rep. 2016 Sep 2;6:32565. doi: 10.1038/srep32565 (PMC5009317; doi:10.1038/srep32565)
Supplement: Supplementary Information [file srep32565-s1.pdf]

Supplementary information:

Supplementary information Figures S1-S4 and Table S1-S5.

Efficient Production of Gene-Modified Mice using *Staphylococcus aureus* Cas9

Xiya Zhang[1,2,#], Puping Liang[1,2,3,#], Chenhui Ding[3,#], Zhen Zhang[2], Jianwen Zhou[4], Xiaowei Xie[2], Rui Huang[2], Ying Sun[2], Hongwei Sun[2], Jinran Zhang[2], Yanwen Xu[3], Zhou Songyang[1,2,3,*], Junjiu Huang[1,2,3,*]

[1]State Key Laboratory of Ophthalmology, Zhongshan Ophthalmic Center, Sun Yat-sen University, Guangzhou, 510275, China.

[2]Key Laboratory of Gene Engineering of the Ministry of Education, Guangzhou Key Laboratory of Healthy Aging Research and State Key Laboratory of Biocontrol, SYSU-BCM Joint Research Center, School of Life Sciences, Sun Yat-sen University, Guangzhou, 510275, China

[3]Key Laboratory of Reproductive Medicine of Guangdong Province, School of Life Sciences and the First Affiliated Hospital, Sun Yat-sen University, Guangzhou, 510275, China

[4]Department of Pathology, First Affiliated Hospital, Sun Yat-sen University，Guangzhou, 510080, China

Supplementary information, Figure S1

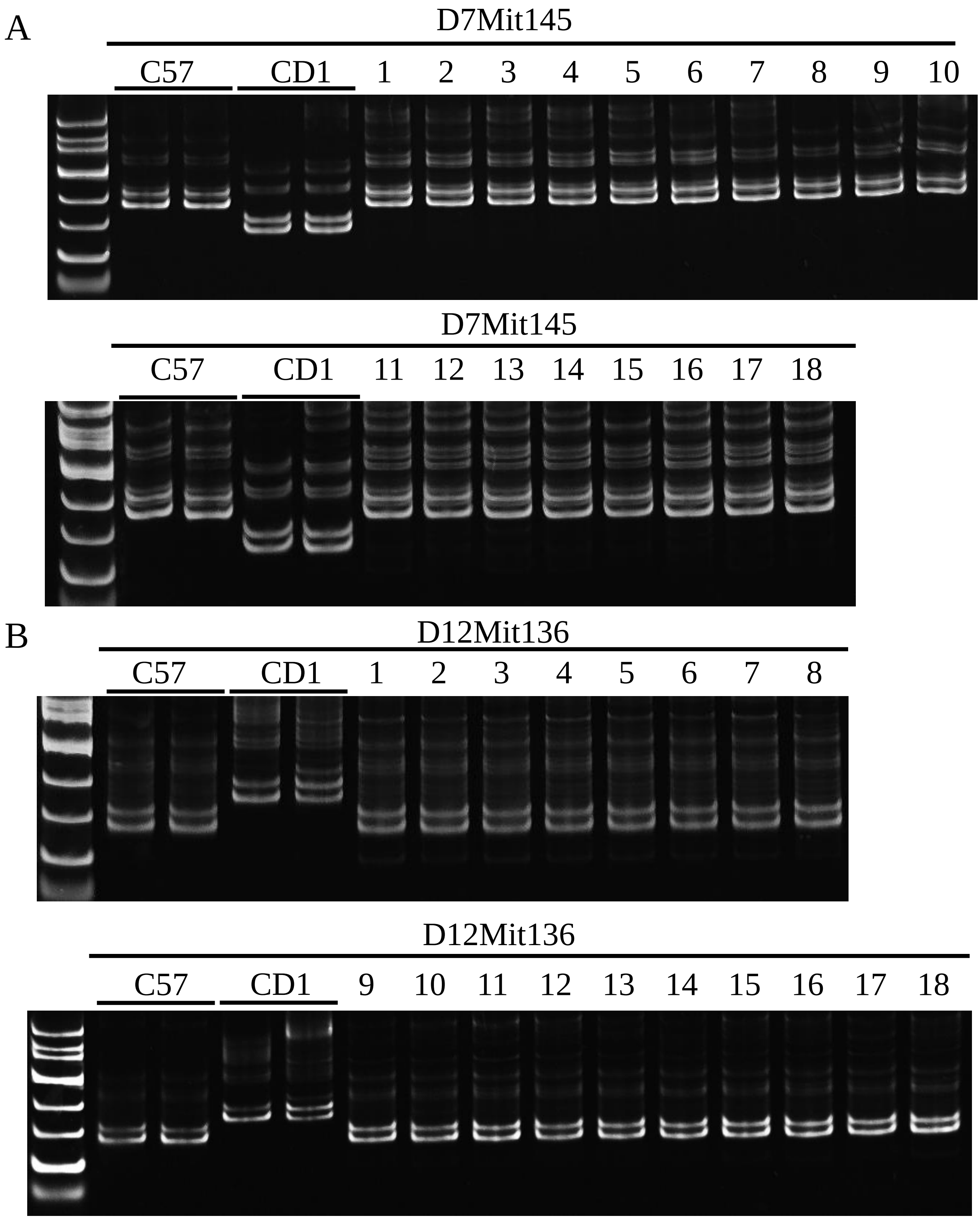

Microsatellite analysis confirmed that the albino and mosaic founders were C57BL/6J mice. (A) D7Mit145 sites, from albino and mosaic founders, were amplified by PCR and then electrophoresis on 10% PAGE gel. (B)Microsatellite analysis using D12Mit136 site to confirm the albino and mosaic founders were C57BL/6J.

Supplementary information, Figure S2

Tyr-SaCas9-G1

WT GTGATTTCTTCATAACATCCAAGGATCTGGGATATGACTACAGCTAC

#2
GTGATTTCTTCATAACATCCAAGGA--TGGGATATGACTACAGCTAC
GTGATTTCTTCATAACATCCAAG--------ATATGACTACAGCTAC
GTGATTTCTTCATAACATCCAAGGGATATGGGATATGGGATATGACTACAGCTAC
GTGATTTCTTCATAACATCTGGG--------ATATGACTACAGCTAC
GTGATTTCTTCATAACATCTGGGG--------TATGACTACAGCTAC

#4
GTGATTTCTTCATAACATCCAAGGATCTGGGATATGACTACAGCTAC
GTGATTTCTTCATAACATCCAATTATATCTGGGATATGACTACAGCTAC
GTGATTTCTTCATAACATCCAA-------TGTTATGACTACTATTAC
GTGATTTCTTCATAACATCCAA-------GGTTATGACTACAGCTAC
GTGATTTCTTCATAACATCCAA-------GGATATGACTACAGCTAC
GTGATTTCTTCATAACATCCAA-------GGTTATGACTACAGCTAC

Tyr-SaCas9-G2

WT GTGATTTCTTCATAACATCCAAGGATCTGGGATATGACTACAGCTAC

#1
GTGATTTCTTCATAACATCCAAGGATCTGGGATATGACTACAGCTAC
GTGATTTCTTCATAACATC--------TGGGATATGACTACAGCTAC
GTGATTTCTTCATAACACCCAAGGATCTGGGATATGACTACAGCTAC
GTGATTTCTTCATAACATCCAAGGA—TGGGATATGACTACAGCTAC

#4
GTGATTTCTTCATAACATCCAAGGATATCTGGGATATGACTACAGCTAC
GTGATTTCTTCATAACATCCAAGGAT-------ATGACTACAGCTAC

Tyr-SpCas9-G3

WT TTACCCAGAAGCCAATGCACCTATCGGCCATAACAGAGACTCTT

#1
TTACCCAGAAGCCATCTGGGTAATCGGCCATAACAGAGACTCTTAC
TTACCCAGAAGCC----------------ATAACAGAGACTCTTAC
TTACCCAGAAGCC--------TATCGGCCATAACAGAGACTCTTAC

#4
TTACCCAGAAGCCA----------------TAACAGAGACTCTTAC
TTACCCAGAAGCCAAT--------CGGCCATAACAGAGACTCTTAC
TTACCCAGAAGCCAAT--------CGGCAATAACAGACACGCTTAC
TTACCCAGAAACCA----------------TAACAGAGACTCTTAC

Tyr-SpCas9-G4

WT TTACCCAGAAGCCAATGCACCTATCGGCCATAACAGAGACTCTTAC

#1
TTACCCAGAAGCCAATGCACATATTC-TCATAAGGTACACTCTTAC
TTACCCAGAAGCCAATGCACCTATCG-CCATAACAGAGACTCTTAC
TTACCCAGAAGCCA----------------TAACAGAGACTCTTAC

#3
TTACCCAGAAGCCAATGCACCTAT-------AACAGAGACTCTTAC
TTACCCAGAAGCCAATGCACCTATCGGGCCATAACAGAGACTCTTAC
TTACCCAGAAGCCAATGCACCTATC-------ACGGAAACTCTTAC

The sequence of mutant alleles in *Tyr* mutant founders. PAM sequence and truncated PAM sequence are labeled in green. Mutant base is labeled in red, and inserted base is in purple.

Supplementary information, Figure S3.

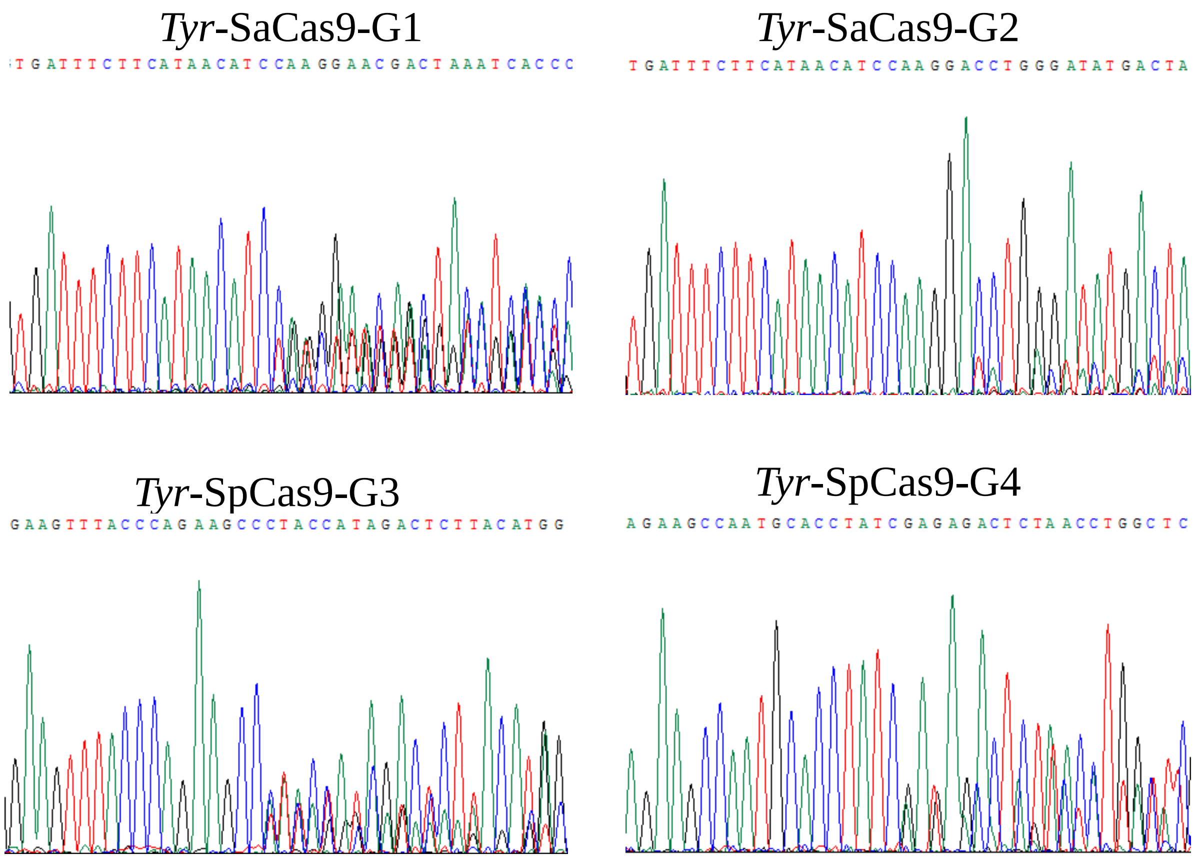

Sanger sequencing to confirm *Tyr* knock-out founder mice. Representative sequencing results showed double peaks near the PAM sequence of target sites.

Supplementary information, Figure S4.

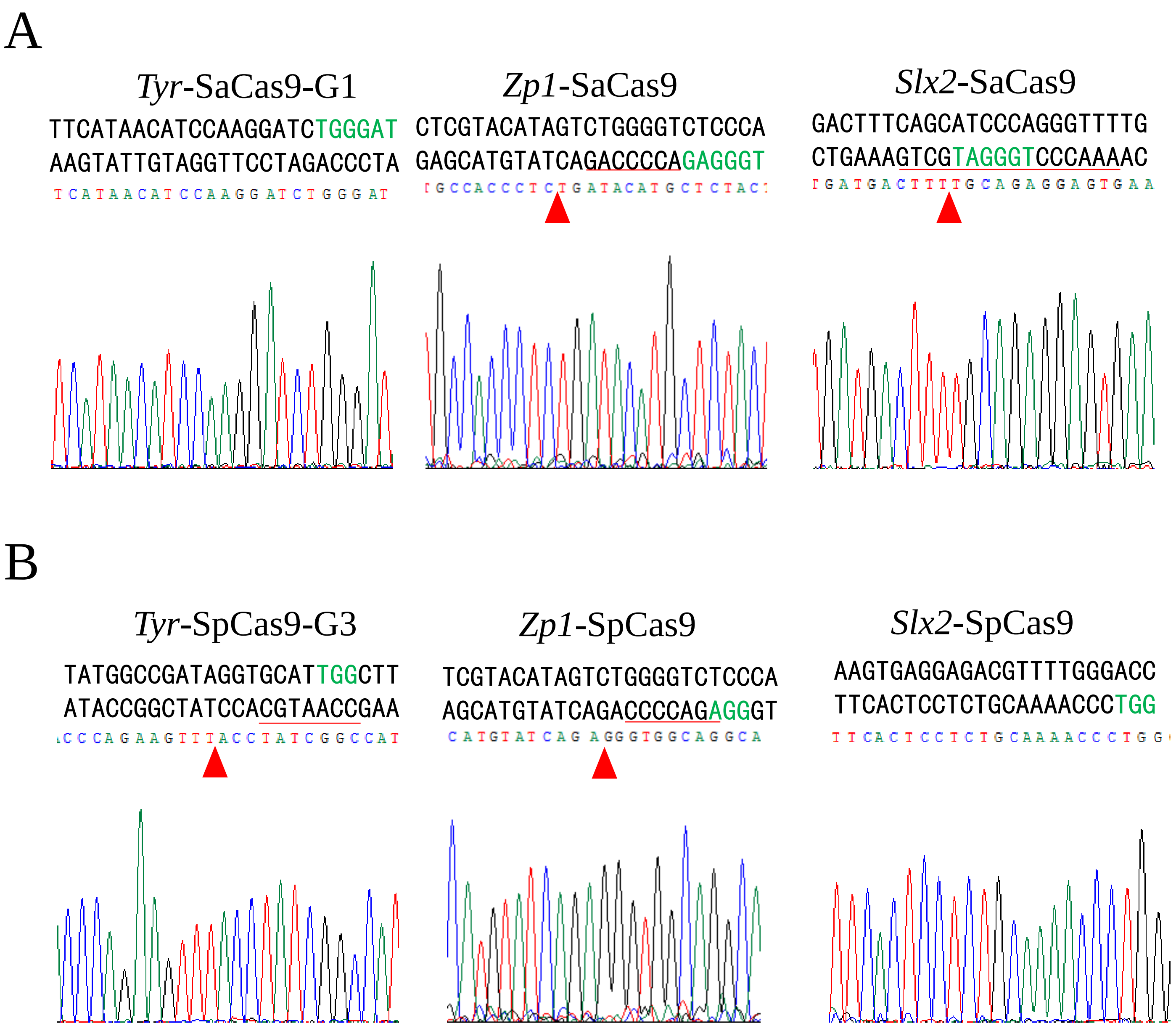

Sanger sequencing to confirm the genotype of multiplex targeted embryos. Sites where some base pairs are deleted are indicated by red triangle, and the deleted base pairs are underlined with red line.

(A) Sanger sequencing to confirm the genotype of #9 embryo of SaCas9.

(B) Sanger sequencing to confirm the genotype of #8 embryo of SpCas9.

Supplementary information Table S1

Single gene disruption efficiency of multiplex gene disruption in mouse embryos by SaCas9 and SpCas9

	Slx2 -targeted embryo No. (%)	*Zp1* -targeted embryo No. (%)	Tyr-targeted embryo No. (%)
SaCas9	17/30 (56.7%)	27/30 (90%)	23/30 (76.7%)
SpCas9	5/37 (13.5%)	29/37 (78.4%)	14/37 (37.8%)

Supplementary information Table S2

Primers used for in vitro transcription of gRNA and saCas9 mRNA

T7-saCas9-FP	TAATACGACTCACTATAGGGAGAATGGCCCCAAAGAAGAAGC
T7-saCas9-RP	TTAGGATCCCTTTTTCTTTTTTGC
SaCas9-gRNA-RP	AAAAATCTCGCCAACAAGTT
SLX2-SaCas9-gRNA-FP	TGTAATACGACTCACTATAGGCCTTCACTCCTCTGCAAAACCCGT TTTAGTACTCTGGAAACAGAATC
ZP1-SaCas9-gRNA-FP	TGTAATACGACTCACTATAGGTAGAGCATGTATCAGACCCCAGTT TTAGTACTCTGGAAACAGAATC
Tyr-SaCas9-G1-FP	TGTAATACGACTCACTATAGGTCTTCATAACATCCAAGGATCGTTTTAGTA CTCTGGAAACAGAATC
Tyr-SaCas9-G2-FP	TGTAATACGACTCACTATAGGCTGTAGTCATATCCCAGATCCGTTTTAGTA CTCTGGAAACAGAATC
SLX2-SpCas9-gRNA-FP	TAGGTTCACTCCTCTGCAAAACCC
SLX2-SpCas9-gRNA-RP	AAACGGGTTTTGCAGAGGAGTGAA
ZP1-SpCas9-gRNA-FP	TAGGAGCATGTATCAGACCCCAG
ZP1-SpCas9-gRNA-RP	AAACCTGGGGTCTGATACATGCT
Tyr-SpCas9-G3-FP	TAGGTTATGGCCGATAGGTGCAT
Tyr-SpCas9-G3-RP	AAACATGCACCTATCGGCCATAA
Tyr-SpCas9-G4-FP	TAGGAGTCTCTGTTATGGCCGAT
Tyr-SpCas9-G4-RP	AAACATCGGCCATAACAGAGACT

Supplementary information Table S3

Primers used for single embryo PCR

ZP1-T7E1-FP	CCTCTGCTCACCCACCTCT
ZP1-T7E1-RP	GCATCCTCAAAGCCCACT
SLX2-T7E1-FP	CACCCCAACAGAGGAAGAAC
SLX2-T7E1-RP	TTTCACAGACTTTTGGCAACTT
Tyr-T7E1-FP	ACACTACTTCTGATGAATGACCTT
Tyr-T7E1-RP	AAATTGGCATAATATAGTTTCTTTA

Supplementary information Table S4

Primers used for microsatellite analysis

D7Mit145-FP	CAGGTGACCTTGGTCATGG
D7Mit145-RP	AGAGCCCAGGGGTTTTAAGA
D12Mit136-FP	TTTAATTTTGAGTGGGTTTGGC
D12Mit136-RP	TTGCTACATGTACACTGATCTCCA

Supplementary information Table S5

Primers for deep sequencing analysis of off-target sites

ZP1-target-1-FP	TAGATCGCAACTGTCTGGCCTCCAACC
ZP1-target-1-RP	CTCTCTATTGCTCACCCACCTCTCTCC
ZP1-target-2-FP	TATCCTCTAACTGTCTGGCCTCCAACC
ZP1-target-2-RP	AGAGTAGATGCTCACCCACCTCTCTCC
ZP1-target-3-FP	GTAAGGAGAACTGTCTGGCCTCCAACC
ZP1-target-3-RP	ACTGCATATGCTCACCCACCTCTCTCC
ZP1-target-4-FP	AAGGAGTAAACTGTCTGGCCTCCAACC
ZP1-target-4-RP	CTAAGCCTTGCTCACCCACCTCTCTCC
ZP1-target-5-FP	CGTCTAATAACTGTCTGGCCTCCAACC
ZP1-target-5-RP	TCTCTCCGTGCTCACCCACCTCTCTCC
ZP1-target-6-FP	TCGACTAGAACTGTCTGGCCTCCAACC
ZP1-target-6-RP	TTCTAGCTTGCTCACCCACCTCTCTCC
ZP1-target-7-FP	CCTAGAGTAACTGTCTGGCCTCCAACC
ZP1-target-7-RP	GCGTAAGATGCTCACCCACCTCTCTCC
ZP1-target-8-FP	CTATTAAGAACTGTCTGGCCTCCAACC
ZP1-target-8-RP	AAGGCTATTGCTCACCCACCTCTCTCC
ZP1-target-9-FP	GAGCCTTAAACTGTCTGGCCTCCAACC
ZP1-target-9-RP	TTATGCGATGCTCACCCACCTCTCTCC
ZP1-target-10-FP	TCGCCTTAAACTGTCTGGCCTCCAACC
ZP1-target-10-RP	CTAGTACGTGCTCACCCACCTCTCTCC

ZP1-sp-OT1-1-FP	TAGATCGCACAGCCAGGGCTATACAGAGAAA
ZP1-sp-OT1-1-RP	CTCTCTATAAGAAAGAAAGAAAGAAAGAAAGCAA
ZP1-sp-OT1-2-FP	TATCCTCTACAGCCAGGGCTATACAGAGAAA
ZP1-sp-OT1-2-RP	AGAGTAGAAAGAAAGAAAGAAAGAAAGAAAGCAA
ZP1-sp-OT1-3-FP	GTAAGGAGACAGCCAGGGCTATACAGAGAAA
ZP1-sp-OT1-3-RP	ACTGCATAAAGAAAGAAAGAAAGAAAGAAAGCAA
ZP1-sp-OT1-4-FP	AAGGAGTAACAGCCAGGGCTATACAGAGAAA
ZP1-sp-OT1-4-RP	CTAAGCCTAAGAAAGAAAGAAAGAAAGAAAGCAA
ZP1-sp-OT1-5-FP	CGTCTAATACAGCCAGGGCTATACAGAGAAA
ZP1-sp-OT1-5-RP	TCTCTCCGAAGAAAGAAAGAAAGAAAGAAAGCAA
ZP1-sp-OT1-6-FP	TCGACTAGACAGCCAGGGCTATACAGAGAAA
ZP1-sp-OT1-6-RP	TTCTAGCTAAGAAAGAAAGAAAGAAAGAAAGCAA
ZP1-sp-OT1-7-FP	CCTAGAGTACAGCCAGGGCTATACAGAGAAA
ZP1-sp-OT1-7-RP	GCGTAAGAAAGAAAGAAAGAAAGAAAGAAAGCAA
ZP1-sp-OT1-8-FP	CTATTAAGACAGCCAGGGCTATACAGAGAAA
ZP1-sp-OT1-8-RP	AAGGCTATAAGAAAGAAAGAAAGAAAGAAAGCAA
ZP1-sp-OT1-9-FP	GAGCCTTAACAGCCAGGGCTATACAGAGAAA
ZP1-sp-OT1-9-RP	TTATGCGAAAGAAAGAAAGAAAGAAAGAAAGCAA
ZP1-sp-OT1-10-FP	TCGCCTTAACAGCCAGGGCTATACAGAGAAA
ZP1-sp-OT1-10-RP	CTAGTACGAAGAAAGAAAGAAAGAAAGAAAGCAA

ZP1-sp-OT2-1-FP	TAGATCGCTAAATTCCTGATGGCTGTCAATG
ZP1-sp-OT2-1-RP	CTCTCTATAGTTCCTTCCAAAATAGCTTGCT
ZP1-sp-OT2-2-FP	TATCCTCTTAAATTCCTGATGGCTGTCAATG

ZP1-sp-OT2-2-RP AGAGTAGAAGTTCCTTCCAAAATAGCTTGCT
ZP1-sp-OT2-3-FP GTAAGGAGTAAATTCCTGATGGCTGTCAATG
ZP1-sp-OT2-3-RP ACTGCATAAGTTCCTTCCAAAATAGCTTGCT
ZP1-sp-OT2-4-FP AAGGAGTATAAATTCCTGATGGCTGTCAATG
ZP1-sp-OT2-4-RP CTAAGCCTAGTTCCTTCCAAAATAGCTTGCT
ZP1-sp-OT2-5-FP CGTCTAATTAAATTCCTGATGGCTGTCAATG
ZP1-sp-OT2-5-RP TCTCTCCGAGTTCCTTCCAAAATAGCTTGCT
ZP1-sp-OT2-6-FP TCGACTAGTAAATTCCTGATGGCTGTCAATG
ZP1-sp-OT2-6-RP TTCTAGCTAGTTCCTTCCAAAATAGCTTGCT
ZP1-sp-OT2-7-FP CCTAGAGTTAAATTCCTGATGGCTGTCAATG
ZP1-sp-OT2-7-RP GCGTAAGAAGTTCCTTCCAAAATAGCTTGCT
ZP1-sp-OT2-8-FP CTATTAAGTAAATTCCTGATGGCTGTCAATG
ZP1-sp-OT2-8-RP AAGGCTATAGTTCCTTCCAAAATAGCTTGCT
ZP1-sp-OT2-9-FP GAGCCTTATAAATTCCTGATGGCTGTCAATG
ZP1-sp-OT2-9-RP TTATGCGAAGTTCCTTCCAAAATAGCTTGCT
ZP1-sp-OT2-10-FP TCGCCTTATAAATTCCTGATGGCTGTCAATG
ZP1-sp-OT2-10-RP CTAGTACGAGTTCCTTCCAAAATAGCTTGCT

ZP1-sp-OT3-1-FP TAGATCGCTGACTTTGAGTTTTTCACTTATTCAC
ZP1-sp-OT3-1-RP CTCTCTATACTAGGAGCTAACCTGCTATGTTG
ZP1-sp-OT3-2-FP TATCCTCTTGACTTTGAGTTTTTCACTTATTCAC
ZP1-sp-OT3-2-RP AGAGTAGACTAGGAGCTAACCTGCTATGTTG
ZP1-sp-OT3-3-FP GTAAGGAGTGACTTTGAGTTTTTCACTTATTCAC
ZP1-sp-OT3-3-RP ACTGCATACTAGGAGCTAACCTGCTATGTTG
ZP1-sp-OT3-4-FP AAGGAGTATGACTTTGAGTTTTTCACTTATTCAC
ZP1-sp-OT3-4-RP CTAAGCCTACTAGGAGCTAACCTGCTATGTTG
ZP1-sp-OT3-5-FP CGTCTAATTGACTTTGAGTTTTTCACTTATTCAC
ZP1-sp-OT3-5-RP TCTCTCCGACTAGGAGCTAACCTGCTATGTTG
ZP1-sp-OT3-6-FP TCGACTAGTGACTTTGAGTTTTTCACTTATTCAC
ZP1-sp-OT3-6-RP TTCTAGCTACTAGGAGCTAACCTGCTATGTTG
ZP1-sp-OT3-7-FP CCTAGAGTTGACTTTGAGTTTTTCACTTATTCAC
ZP1-sp-OT3-7-RP GCGTAAGACTAGGAGCTAACCTGCTATGTTG
ZP1-sp-OT3-8-FP CTATTAAGTGACTTTGAGTTTTTCACTTATTCAC
ZP1-sp-OT3-8-RP AAGGCTATACTAGGAGCTAACCTGCTATGTTG
ZP1-sp-OT3-9-FP GAGCCTTATGACTTTGAGTTTTTCACTTATTCAC
ZP1-sp-OT3-9-RP TTATGCGACTAGGAGCTAACCTGCTATGTTG
ZP1-sp-OT3-10-FP TCGCCTTATGACTTTGAGTTTTTCACTTATTCAC
ZP1-sp-OT3-10-RP CTAGTACGACTAGGAGCTAACCTGCTATGTTG

ZP1-sp-OT4-1-FP TAGATCGCGTGGAAACAAGACTTGGTGTATC
ZP1-sp-OT4-1-RP CTCTCTATGAATTCCTCTTCGTTCTAGGTGATA
ZP1-sp-OT4-2-FP TATCCTCTGTGGAAACAAGACTTGGTGTATC
ZP1-sp-OT4-2-RP AGAGTAGAGAATTCCTCTTCGTTCTAGGTGATA
ZP1-sp-OT4-3-FP GTAAGGAGGTGGAAACAAGACTTGGTGTATC
ZP1-sp-OT4-3-RP ACTGCATAGAATTCCTCTTCGTTCTAGGTGATA
ZP1-sp-OT4-4-FP AAGGAGTAGTGGAAACAAGACTTGGTGTATC
ZP1-sp-OT4-4-RP CTAAGCCTGAATTCCTCTTCGTTCTAGGTGATA
ZP1-sp-OT4-5-FP CGTCTAATGTGGAAACAAGACTTGGTGTATC

ZP1-sp-OT4-5-RP		TCTCTCCGGAATTCCTCTTCGTTCTAGGTGATA
ZP1-sp-OT4-6-FP	TCGACTAGGTGGAAACAAGACTTGGTGTATC	
ZP1-sp-OT4-6-RP		TTCTAGCTGAATTCCTCTTCGTTCTAGGTGATA
ZP1-sp-OT4-7-FP	CCTAGAGTGTGGAAACAAGACTTGGTGTATC	
ZP1-sp-OT4-7-RP		GCGTAAGAGAATTCCTCTTCGTTCTAGGTGATA
ZP1-sp-OT4-8-FP	CTATTAAGGTGGAAACAAGACTTGGTGTATC	
ZP1-sp-OT4-8-RP		AAGGCTATGAATTCCTCTTCGTTCTAGGTGATA
ZP1-sp-OT4-9-FP	GAGCCTTAGTGGAAACAAGACTTGGTGTATC	
ZP1-sp-OT4-9-RP		TTATGCGAGAATTCCTCTTCGTTCTAGGTGATA
ZP1-sp-OT4-10-FP	TCGCCTTAGTGGAAACAAGACTTGGTGTATC	
ZP1-sp-OT4-10-RP		CTAGTACGGAATTCCTCTTCGTTCTAGGTGATA
ZP1-sp-OT5-1-FP	TAGATCGCCATTAGTTCCCATCTCTGGTCCTT	
ZP1-sp-OT5-1-RP		CTCTCTATGAAATCTGGTCGTTCAGGTTGA
ZP1-sp-OT5-2-FP	TATCCTCTCATTAGTTCCCATCTCTGGTCCTT	
ZP1-sp-OT5-2-RP		AGAGTAGAGAAATCTGGTCGTTCAGGTTGA
ZP1-sp-OT5-3-FP	GTAAGGAGCATTAGTTCCCATCTCTGGTCCTT	
ZP1-sp-OT5-3-RP		ACTGCATAGAAATCTGGTCGTTCAGGTTGA
ZP1-sp-OT5-4-FP	AAGGAGTACATTAGTTCCCATCTCTGGTCCTT	
ZP1-sp-OT5-4-RP		CTAAGCCTGAAATCTGGTCGTTCAGGTTGA
ZP1-sp-OT5-5-FP	CGTCTAATCATTAGTTCCCATCTCTGGTCCTT	
ZP1-sp-OT5-5-RP		TCTCTCCGGAAATCTGGTCGTTCAGGTTGA
ZP1-sp-OT5-6-FP	TCGACTAGCATTAGTTCCCATCTCTGGTCCTT	
ZP1-sp-OT5-6-RP		TTCTAGCTGAAATCTGGTCGTTCAGGTTGA
ZP1-sp-OT5-7-FP	CCTAGAGTCATTAGTTCCCATCTCTGGTCCTT	
ZP1-sp-OT5-7-RP		GCGTAAGAGAAATCTGGTCGTTCAGGTTGA
ZP1-sp-OT5-8-FP	CTATTAAGCATTAGTTCCCATCTCTGGTCCTT	
ZP1-sp-OT5-8-RP		AAGGCTATGAAATCTGGTCGTTCAGGTTGA
ZP1-sp-OT5-9-FP	GAGCCTTACATTAGTTCCCATCTCTGGTCCTT	
ZP1-sp-OT5-9-RP		TTATGCGAGAAATCTGGTCGTTCAGGTTGA
ZP1-sp-OT5-10-FP	TCGCCTTACATTAGTTCCCATCTCTGGTCCTT	
ZP1-sp-OT5-10-RP		CTAGTACGGAAATCTGGTCGTTCAGGTTGA
ZP1-sp-OT6-1-FP	TAGATCGCAATGAGCACAGAGGAAATGTTTAT	
ZP1-sp-OT6-1-RP		CTCTCTATCTTGGTTTTGTTCTTCAAAGTCTC
ZP1-sp-OT6-2-FP	TATCCTCTAATGAGCACAGAGGAAATGTTTAT	
ZP1-sp-OT6-2-RP		AGAGTAGACTTGGTTTTGTTCTTCAAAGTCTC
ZP1-sp-OT6-3-FP	GTAAGGAGAATGAGCACAGAGGAAATGTTTAT	
ZP1-sp-OT6-3-RP		ACTGCATACTTGGTTTTGTTCTTCAAAGTCTC
ZP1-sp-OT6-4-FP	AAGGAGTAAATGAGCACAGAGGAAATGTTTAT	
ZP1-sp-OT6-4-RP		CTAAGCCTCTTGGTTTTGTTCTTCAAAGTCTC
ZP1-sp-OT6-5-FP	CGTCTAATAATGAGCACAGAGGAAATGTTTAT	
ZP1-sp-OT6-5-RP		TCTCTCCGCTTGGTTTTGTTCTTCAAAGTCTC
ZP1-sp-OT6-6-FP	TCGACTAGAATGAGCACAGAGGAAATGTTTAT	
ZP1-sp-OT6-6-RP		TTCTAGCTCTTGGTTTTGTTCTTCAAAGTCTC
ZP1-sp-OT6-7-FP	CCTAGAGTAATGAGCACAGAGGAAATGTTTAT	
ZP1-sp-OT6-7-RP		GCGTAAGACTTGGTTTTGTTCTTCAAAGTCTC
ZP1-sp-OT6-8-FP	CTATTAAGAATGAGCACAGAGGAAATGTTTAT	

ZP1-sp-OT6-8-RP		AAGGCTATCTTGGTTTTGTTCTTCAAAGTCTC
ZP1-sp-OT6-9-FP	GAGCCTTAAATGAGCACAGAGGAAATGTTTAT	
ZP1-sp-OT6-9-RP		TTATGCGACTTGGTTTTGTTCTTCAAAGTCTC
ZP1-sp-OT6-10-FP	TCGCCTTAAATGAGCACAGAGGAAATGTTTAT	
ZP1-sp-OT6-10-RP		CTAGTACGCTTGGTTTTGTTCTTCAAAGTCTC
ZP1-sp-OT7-1-FP	TAGATCGCTGCCAGTATGTAAATGGGTTCTAT	
ZP1-sp-OT7-1-RP		CTCTCTATACTGTCTTTCAGTGTGAGCCAAG
ZP1-sp-OT7-2-FP	TATCCTCTTGCCAGTATGTAAATGGGTTCTAT	
ZP1-sp-OT7-2-RP		AGAGTAGAACTGTCTTTCAGTGTGAGCCAAG
ZP1-sp-OT7-3-FP	GTAAGGAGTGCCAGTATGTAAATGGGTTCTAT	
ZP1-sp-OT7-3-RP		ACTGCATAACTGTCTTTCAGTGTGAGCCAAG
ZP1-sp-OT7-4-FP	AAGGAGTATGCCAGTATGTAAATGGGTTCTAT	
ZP1-sp-OT7-4-RP		CTAAGCCTACTGTCTTTCAGTGTGAGCCAAG
ZP1-sp-OT7-5-FP	CGTCTAATTGCCAGTATGTAAATGGGTTCTAT	
ZP1-sp-OT7-5-RP		TCTCTCCGACTGTCTTTCAGTGTGAGCCAAG
ZP1-sp-OT7-6-FP	TCGACTAGTGCCAGTATGTAAATGGGTTCTAT	
ZP1-sp-OT7-6-RP		TTCTAGCTACTGTCTTTCAGTGTGAGCCAAG
ZP1-sp-OT7-7-FP	CCTAGAGTTGCCAGTATGTAAATGGGTTCTAT	
ZP1-sp-OT7-7-RP		GCGTAAGAACTGTCTTTCAGTGTGAGCCAAG
ZP1-sp-OT7-8-FP	CTATTAAGTGCCAGTATGTAAATGGGTTCTAT	
ZP1-sp-OT7-8-RP		AAGGCTATACTGTCTTTCAGTGTGAGCCAAG
ZP1-sp-OT7-9-FP	GAGCCTTATGCCAGTATGTAAATGGGTTCTAT	
ZP1-sp-OT7-9-RP		TTATGCGAACTGTCTTTCAGTGTGAGCCAAG
ZP1-sp-OT7-10-FP	TCGCCTTATGCCAGTATGTAAATGGGTTCTAT	
ZP1-sp-OT7-10-RP		CTAGTACGACTGTCTTTCAGTGTGAGCCAAG
ZP1-sp-OT8-1-FP	TAGATCGCCATCACCCCAATAGATGTTTATGC	
ZP1-sp-OT8-1-RP		CTCTCTATGGTCATTTGTGAAGTGTTTTGTCC
ZP1-sp-OT8-2-FP	TATCCTCTCATCACCCCAATAGATGTTTATGC	
ZP1-sp-OT8-2-RP		AGAGTAGAGGTCATTTGTGAAGTGTTTTGTCC
ZP1-sp-OT8-3-FP	GTAAGGAGCATCACCCCAATAGATGTTTATGC	
ZP1-sp-OT8-3-RP		ACTGCATAGGTCATTTGTGAAGTGTTTTGTCC
ZP1-sp-OT8-4-FP	AAGGAGTACATCACCCCAATAGATGTTTATGC	
ZP1-sp-OT8-4-RP		CTAAGCCTGGTCATTTGTGAAGTGTTTTGTCC
ZP1-sp-OT8-5-FP	CGTCTAATCATCACCCCAATAGATGTTTATGC	
ZP1-sp-OT8-5-RP		TCTCTCCGGGTCATTTGTGAAGTGTTTTGTCC
ZP1-sp-OT8-6-FP	TCGACTAGCATCACCCCAATAGATGTTTATGC	
ZP1-sp-OT8-6-RP		TTCTAGCTGGTCATTTGTGAAGTGTTTTGTCC
ZP1-sp-OT8-7-FP	CCTAGAGTCATCACCCCAATAGATGTTTATGC	
ZP1-sp-OT8-7-RP		GCGTAAGAGGTCATTTGTGAAGTGTTTTGTCC
ZP1-sp-OT8-8-FP	CTATTAAGCATCACCCCAATAGATGTTTATGC	
ZP1-sp-OT8-8-RP		AAGGCTATGGTCATTTGTGAAGTGTTTTGTCC
ZP1-sp-OT8-9-FP	GAGCCTTACATCACCCCAATAGATGTTTATGC	
ZP1-sp-OT8-9-RP		TTATGCGAGGTCATTTGTGAAGTGTTTTGTCC
ZP1-sp-OT8-10-FP	TCGCCTTACATCACCCCAATAGATGTTTATGC	
ZP1-sp-OT8-10-RP		CTAGTACGGGTCATTTGTGAAGTGTTTTGTCC

ZP1-sp-OT9-1-FP	TAGATCGCTTAACTTAGCTGCATTCTGCTTCC	
ZP1-sp-OT9-1-RP		CTCTCTATCTTCATCTTTTGGCTCACTTTTCA
ZP1-sp-OT9-2-FP	TATCCTCTTTAACTTAGCTGCATTCTGCTTCC	
ZP1-sp-OT9-2-RP		AGAGTAGACTTCATCTTTTGGCTCACTTTTCA
ZP1-sp-OT9-3-FP	GTAAGGAGTTAACTTAGCTGCATTCTGCTTCC	
ZP1-sp-OT9-3-RP		ACTGCATACTTCATCTTTTGGCTCACTTTTCA
ZP1-sp-OT9-4-FP	AAGGAGTATTAACTTAGCTGCATTCTGCTTCC	
ZP1-sp-OT9-4-RP		CTAAGCCTCTTCATCTTTTGGCTCACTTTTCA
ZP1-sp-OT9-5-FP	CGTCTAATTTAACTTAGCTGCATTCTGCTTCC	
ZP1-sp-OT9-5-RP		TCTCTCCGCTTCATCTTTTGGCTCACTTTTCA
ZP1-sp-OT9-6-FP	TCGACTAGTTAACTTAGCTGCATTCTGCTTCC	
ZP1-sp-OT9-6-RP		TTCTAGCTCTTCATCTTTTGGCTCACTTTTCA
ZP1-sp-OT9-7-FP	CCTAGAGTTTAACTTAGCTGCATTCTGCTTCC	
ZP1-sp-OT9-7-RP		GCGTAAGACTTCATCTTTTGGCTCACTTTTCA
ZP1-sp-OT9-8-FP	CTATTAAGTTAACTTAGCTGCATTCTGCTTCC	
ZP1-sp-OT9-8-RP		AAGGCTATCTTCATCTTTTGGCTCACTTTTCA
ZP1-sp-OT9-9-FP	GAGCCTTATTAACTTAGCTGCATTCTGCTTCC	
ZP1-sp-OT9-9-RP		TTATGCGACTTCATCTTTTGGCTCACTTTTCA
ZP1-sp-OT9-10-FP	TCGCCTTATTAACTTAGCTGCATTCTGCTTCC	
ZP1-sp-OT9-10-RP		CTAGTACGCTTCATCTTTTGGCTCACTTTTCA
ZP1-sp-OT10-1-FP	TAGATCGCCAGAATTTAAACAGGGGAGGAA	
ZP1-sp-OT10-1-RP		CTCTCTATCAAAAACAAAAACACAGGGGAC
ZP1-sp-OT10-2-FP	TATCCTCTCAGAATTTAAACAGGGGAGGAA	
ZP1-sp-OT10-2-RP		AGAGTAGACAAAAACAAAAACACAGGGGAC
ZP1-sp-OT10-3-FP	GTAAGGAGCAGAATTTAAACAGGGGAGGAA	
ZP1-sp-OT10-3-RP		ACTGCATACAAAAACAAAAACACAGGGGAC
ZP1-sp-OT10-4-FP	AAGGAGTACAGAATTTAAACAGGGGAGGAA	
ZP1-sp-OT10-4-RP		CTAAGCCTCAAAAACAAAAACACAGGGGAC
ZP1-sp-OT10-5-FP	CGTCTAATCAGAATTTAAACAGGGGAGGAA	
ZP1-sp-OT10-5-RP		TCTCTCCGCAAAAACAAAAACACAGGGGAC
ZP1-sp-OT10-6-FP	TCGACTAGCAGAATTTAAACAGGGGAGGAA	
ZP1-sp-OT10-6-RP		TTCTAGCTCAAAAACAAAAACACAGGGGAC
ZP1-sp-OT10-7-FP	CCTAGAGTCAGAATTTAAACAGGGGAGGAA	
ZP1-sp-OT10-7-RP		GCGTAAGACAAAAACAAAAACACAGGGGAC
ZP1-sp-OT10-8-FP	CTATTAAGCAGAATTTAAACAGGGGAGGAA	
ZP1-sp-OT10-8-RP		AAGGCTATCAAAAACAAAAACACAGGGGAC
ZP1-sp-OT10-9-FP	GAGCCTTACAGAATTTAAACAGGGGAGGAA	
ZP1-sp-OT10-9-RP		TTATGCGACAAAAACAAAAACACAGGGGAC
ZP1-sp-OT10-10-FP	TCGCCTTACAGAATTTAAACAGGGGAGGAA	
ZP1-sp-OT10-10-RP		CTAGTACGCAAAAACAAAAACACAGGGGAC
ZP1-target-11-FP	TTCTGCCTAACTGTCTGGCCTCCAACC	
ZP1-target-11-RP		GCTCAGGATGCTCACCCACCTCTCTCC
ZP1-target-12-FP	AGGAGTCCAACTGTCTGGCCTCCAACC	
ZP1-target-12-RP		CATGCCTATGCTCACCCACCTCTCTCC
ZP1-target-13-FP	GTAGAGAGAACTGTCTGGCCTCCAACC	
ZP1-target-13-RP		CCTCTCTGTGCTCACCCACCTCTCTCC

ZP1-target-14-FP	AGCGTAGCAACTGTCTGGCCTCCAACC	
ZP1-target-14-RP		CAGCCTCGTGCTCACCCACCTCTCTCC
ZP1-target-15-FP	TGCCTCTTAACTGTCTGGCCTCCAACC	
ZP1-target-15-RP		TCCTCTACTGCTCACCCACCTCTCTCC
ZP1-target-16-FP	TCATGAGCAACTGTCTGGCCTCCAACC	
ZP1-target-16-RP		CCTGAGATTGCTCACCCACCTCTCTCC
ZP1-target-17-FP	TAGCGAGTAACTGTCTGGCCTCCAACC	
ZP1-target-17-RP		GTAGCTCCTGCTCACCCACCTCTCTCC
ZP1-target-18-FP	TACTACGCAACTGTCTGGCCTCCAACC	
ZP1-target-18-RP		AGGCTCCGTGCTCACCCACCTCTCTCC
ZP1-target-19-FP	GCAGCGTAAACTGTCTGGCCTCCAACC	
ZP1-target-19-RP		CTGCGCATTGCTCACCCACCTCTCTCC
ZP1-target-20-FP	GAGCGCTAAACTGTCTGGCCTCCAACC	
ZP1-target-20-RP		CGCTCAGTTGCTCACCCACCTCTCTCC

ZP1-sa-OT1-11-FP	TTCTGCCTCCTTTGAAGGGTGTCAGGAAA	
ZP1-sa-OT1-11-RP		GCTCAGGAGCTGGCATCGGGTATCGT
ZP1-sa-OT1-12-FP	AGGAGTCCCCTTTGAAGGGTGTCAGGAAA	
ZP1-sa-OT1-12-RP		CATGCCTAGCTGGCATCGGGTATCGT
ZP1-sa-OT1-13-FP	GTAGAGAGCCTTTGAAGGGTGTCAGGAAA	
ZP1-sa-OT1-13-RP		CCTCTCTGGCTGGCATCGGGTATCGT
ZP1-sa-OT1-14-FP	AGCGTAGCCCTTTGAAGGGTGTCAGGAAA	
ZP1-sa-OT1-14-RP		CAGCCTCGGCTGGCATCGGGTATCGT
ZP1-sa-OT1-15-FP	TGCCTCTTCCTTTGAAGGGTGTCAGGAAA	
ZP1-sa-OT1-15-RP		TCCTCTACGCTGGCATCGGGTATCGT
ZP1-sa-OT1-16-FP	TCATGAGCCCTTTGAAGGGTGTCAGGAAA	
ZP1-sa-OT1-16-RP		CCTGAGATGCTGGCATCGGGTATCGT
ZP1-sa-OT1-17-FP	TAGCGAGTCCTTTGAAGGGTGTCAGGAAA	
ZP1-sa-OT1-17-RP		GTAGCTCCGCTGGCATCGGGTATCGT
ZP1-sa-OT1-18-FP	TACTACGCCCTTTGAAGGGTGTCAGGAAA	
ZP1-sa-OT1-18-RP		AGGCTCCGGCTGGCATCGGGTATCGT
ZP1-sa-OT1-19-FP	GCAGCGTACCTTTGAAGGGTGTCAGGAAA	
ZP1-sa-OT1-19-RP		CTGCGCATGCTGGCATCGGGTATCGT
ZP1-sa-OT1-20-FP	GAGCGCTACCTTTGAAGGGTGTCAGGAAA	
ZP1-sa-OT1-20-RP		CGCTCAGTGCTGGCATCGGGTATCGT

ZP1-sa-OT2-11-FP	TTCTGCCTTTTGTTTATTGCCTTTCAGC	
ZP1-sa-OT2-11-RP		GCTCAGGAAATGGAGGGGTAGTGGTTTG
ZP1-sa-OT2-12-FP	AGGAGTCCTTTGTTTATTGCCTTTCAGC	
ZP1-sa-OT2-12-RP		CATGCCTAAATGGAGGGGTAGTGGTTTG
ZP1-sa-OT2-13-FP	GTAGAGAGTTTGTTTATTGCCTTTCAGC	
ZP1-sa-OT2-13-RP		CCTCTCTGAATGGAGGGGTAGTGGTTTG
ZP1-sa-OT2-14-FP	AGCGTAGCTTTGTTTATTGCCTTTCAGC	
ZP1-sa-OT2-14-RP		CAGCCTCGAATGGAGGGGTAGTGGTTTG
ZP1-sa-OT2-15-FP	TGCCTCTTTTTGTTTATTGCCTTTCAGC	
ZP1-sa-OT2-15-RP		TCCTCTACAATGGAGGGGTAGTGGTTTG
ZP1-sa-OT2-16-FP	TCATGAGCTTTGTTTATTGCCTTTCAGC	
ZP1-sa-OT2-16-RP		CCTGAGATAATGGAGGGGTAGTGGTTTG

ZP1-sa-OT2-17-FP	TAGCGAGTTTTGTTTATTGCCTTTCAGC
ZP1-sa-OT2-17-RP	GTAGCTCCAATGGAGGGGTAGTGGTTTG
ZP1-sa-OT2-18-FP	TACTACGCTTTGTTTATTGCCTTTCAGC
ZP1-sa-OT2-18-RP	AGGCTCCGAATGGAGGGGTAGTGGTTTG
ZP1-sa-OT2-19-FP	GCAGCGTATTTGTTTATTGCCTTTCAGC
ZP1-sa-OT2-19-RP	CTGCGCATAATGGAGGGGTAGTGGTTTG
ZP1-sa-OT2-20-FP	GAGCGCTATTTGTTTATTGCCTTTCAGC
ZP1-sa-OT2-20-RP	CGCTCAGTAATGGAGGGGTAGTGGTTTG

ZP1-sa-OT3-11-FP	TTCTGCCTCCTTGTGAATGAGGTTGAATGT
ZP1-sa-OT3-11-RP	GCTCAGGAAAGTGTCTTGTTTCCTCCTTTG
ZP1-sa-OT3-12-FP	AGGAGTCCCCTTGTGAATGAGGTTGAATGT
ZP1-sa-OT3-12-RP	CATGCCTAAAGTGTCTTGTTTCCTCCTTTG
ZP1-sa-OT3-13-FP	GTAGAGAGCCTTGTGAATGAGGTTGAATGT
ZP1-sa-OT3-13-RP	CCTCTCTGAAGTGTCTTGTTTCCTCCTTTG
ZP1-sa-OT3-14-FP	AGCGTAGCCCTTGTGAATGAGGTTGAATGT
ZP1-sa-OT3-14-RP	CAGCCTCGAAGTGTCTTGTTTCCTCCTTTG
ZP1-sa-OT3-15-FP	TGCCTCTTCCTTGTGAATGAGGTTGAATGT
ZP1-sa-OT3-15-RP	TCCTCTACAAGTGTCTTGTTTCCTCCTTTG
ZP1-sa-OT3-16-FP	TCATGAGCCCTTGTGAATGAGGTTGAATGT
ZP1-sa-OT3-16-RP	CCTGAGATAAGTGTCTTGTTTCCTCCTTTG
ZP1-sa-OT3-17-FP	TAGCGAGTCCTTGTGAATGAGGTTGAATGT
ZP1-sa-OT3-17-RP	GTAGCTCCAAGTGTCTTGTTTCCTCCTTTG
ZP1-sa-OT3-18-FP	TACTACGCCCTTGTGAATGAGGTTGAATGT
ZP1-sa-OT3-18-RP	AGGCTCCGAAGTGTCTTGTTTCCTCCTTTG
ZP1-sa-OT3-19-FP	GCAGCGTACCTTGTGAATGAGGTTGAATGT
ZP1-sa-OT3-19-RP	CTGCGCATAAGTGTCTTGTTTCCTCCTTTG
ZP1-sa-OT3-20-FP	GAGCGCTACCTTGTGAATGAGGTTGAATGT
ZP1-sa-OT3-20-RP	CGCTCAGTAAGTGTCTTGTTTCCTCCTTTG

ZP1-sa-OT4-11-FP	TTCTGCCTCATCTCCACATAAGGAGACAAA
ZP1-sa-OT4-11-RP	GCTCAGGATTTCCCTTTCTGAAGATTTACC
ZP1-sa-OT4-12-FP	AGGAGTCCCATCTCCACATAAGGAGACAAA
ZP1-sa-OT4-12-RP	CATGCCTATTTCCCTTTCTGAAGATTTACC
ZP1-sa-OT4-13-FP	GTAGAGAGCATCTCCACATAAGGAGACAAA
ZP1-sa-OT4-13-RP	CCTCTCTGTTTCCCTTTCTGAAGATTTACC
ZP1-sa-OT4-14-FP	AGCGTAGCCATCTCCACATAAGGAGACAAA
ZP1-sa-OT4-14-RP	CAGCCTCGTTTCCCTTTCTGAAGATTTACC
ZP1-sa-OT4-15-FP	TGCCTCTTCATCTCCACATAAGGAGACAAA
ZP1-sa-OT4-15-RP	TCCTCTACTTTCCCTTTCTGAAGATTTACC
ZP1-sa-OT4-16-FP	TCATGAGCCATCTCCACATAAGGAGACAAA
ZP1-sa-OT4-16-RP	CCTGAGATTTTCCCTTTCTGAAGATTTACC
ZP1-sa-OT4-17-FP	TAGCGAGTCATCTCCACATAAGGAGACAAA
ZP1-sa-OT4-17-RP	GTAGCTCCTTTCCCTTTCTGAAGATTTACC
ZP1-sa-OT4-18-FP	TACTACGCCATCTCCACATAAGGAGACAAA
ZP1-sa-OT4-18-RP	AGGCTCCGTTTCCCTTTCTGAAGATTTACC
ZP1-sa-OT4-19-FP	GCAGCGTACATCTCCACATAAGGAGACAAA
ZP1-sa-OT4-19-RP	CTGCGCATTTTCCCTTTCTGAAGATTTACC

ZP1-sa-OT4-20-FP	GAGCGCTACATCTCCACATAAGGAGACAAA	
ZP1-sa-OT4-20-RP		CGCTCAGTTTTCCCTTTCTGAAGATTTACC

ZP1-sa-OT5-11-FP	TTCTGCCTCAGGCAGATAGCAAGTTCAAGG	
ZP1-sa-OT5-11-RP		GCTCAGGAACCAGCAAACACGAGTCAGATAA
ZP1-sa-OT5-12-FP	AGGAGTCCCAGGCAGATAGCAAGTTCAAGG	
ZP1-sa-OT5-12-RP		CATGCCTAACCAGCAAACACGAGTCAGATAA
ZP1-sa-OT5-13-FP	GTAGAGAGCAGGCAGATAGCAAGTTCAAGG	
ZP1-sa-OT5-13-RP		CCTCTCTGACCAGCAAACACGAGTCAGATAA
ZP1-sa-OT5-14-FP	AGCGTAGCCAGGCAGATAGCAAGTTCAAGG	
ZP1-sa-OT5-14-RP		CAGCCTCGACCAGCAAACACGAGTCAGATAA
ZP1-sa-OT5-15-FP	TGCCTCTTCAGGCAGATAGCAAGTTCAAGG	
ZP1-sa-OT5-15-RP		TCCTCTACACCAGCAAACACGAGTCAGATAA
ZP1-sa-OT5-16-FP	TCATGAGCCAGGCAGATAGCAAGTTCAAGG	
ZP1-sa-OT5-16-RP		CCTGAGATACCAGCAAACACGAGTCAGATAA
ZP1-sa-OT5-17-FP	TAGCGAGTCAGGCAGATAGCAAGTTCAAGG	
ZP1-sa-OT5-17-RP		GTAGCTCCACCAGCAAACACGAGTCAGATAA
ZP1-sa-OT5-18-FP	TACTACGCCAGGCAGATAGCAAGTTCAAGG	
ZP1-sa-OT5-18-RP		AGGCTCCGACCAGCAAACACGAGTCAGATAA
ZP1-sa-OT5-19-FP	GCAGCGTACAGGCAGATAGCAAGTTCAAGG	
ZP1-sa-OT5-19-RP		CTGCGCATACCAGCAAACACGAGTCAGATAA
ZP1-sa-OT5-20-FP	GAGCGCTACAGGCAGATAGCAAGTTCAAGG	
ZP1-sa-OT5-20-RP		CGCTCAGTACCAGCAAACACGAGTCAGATAA

ZP1-sa-OT6-11-FP	TTCTGCCTCCAGGTTTCTAGTGCCAAGTCA	
ZP1-sa-OT6-11-RP		GCTCAGGAAACAGGTTATGAAAAGCGGACA
ZP1-sa-OT6-12-FP	AGGAGTCCCCAGGTTTCTAGTGCCAAGTCA	
ZP1-sa-OT6-12-RP		CATGCCTAAACAGGTTATGAAAAGCGGACA
ZP1-sa-OT6-13-FP	GTAGAGAGCCAGGTTTCTAGTGCCAAGTCA	
ZP1-sa-OT6-13-RP		CCTCTCTGAACAGGTTATGAAAAGCGGACA
ZP1-sa-OT6-14-FP	AGCGTAGCCCAGGTTTCTAGTGCCAAGTCA	
ZP1-sa-OT6-14-RP		CAGCCTCGAACAGGTTATGAAAAGCGGACA
ZP1-sa-OT6-15-FP	TGCCTCTTCCAGGTTTCTAGTGCCAAGTCA	
ZP1-sa-OT6-15-RP		TCCTCTACAACAGGTTATGAAAAGCGGACA
ZP1-sa-OT6-16-FP	TCATGAGCCCAGGTTTCTAGTGCCAAGTCA	
ZP1-sa-OT6-16-RP		CCTGAGATAACAGGTTATGAAAAGCGGACA
ZP1-sa-OT6-17-FP	TAGCGAGTCCAGGTTTCTAGTGCCAAGTCA	
ZP1-sa-OT6-17-RP		GTAGCTCCAACAGGTTATGAAAAGCGGACA
ZP1-sa-OT6-18-FP	TACTACGCCCAGGTTTCTAGTGCCAAGTCA	
ZP1-sa-OT6-18-RP		AGGCTCCGAACAGGTTATGAAAAGCGGACA
ZP1-sa-OT6-19-FP	GCAGCGTACCAGGTTTCTAGTGCCAAGTCA	
ZP1-sa-OT6-19-RP		CTGCGCATAACAGGTTATGAAAAGCGGACA
ZP1-sa-OT6-20-FP	GAGCGCTACCAGGTTTCTAGTGCCAAGTCA	
ZP1-sa-OT6-20-RP		CGCTCAGTAACAGGTTATGAAAAGCGGACA

ZP1-sa-OT7-11-FP	TTCTGCCTTAGACACCACGCAACCACC	
ZP1-sa-OT7-11-RP		GCTCAGGATCCAAAGGGTTTGTCATCAG
ZP1-sa-OT7-12-FP	AGGAGTCCTAGACACCACGCAACCACC	

ZP1-sa-OT7-12-RP		CATGCCTATCCAAAGGGTTTGTCATCAG
ZP1-sa-OT7-13-FP	GTAGAGAGTAGACACCACGCAACCACC	
ZP1-sa-OT7-13-RP		CCTCTCTGTCCAAAGGGTTTGTCATCAG
ZP1-sa-OT7-14-FP	AGCGTAGCTAGACACCACGCAACCACC	
ZP1-sa-OT7-14-RP		CAGCCTCGTCCAAAGGGTTTGTCATCAG
ZP1-sa-OT7-15-FP	TGCCTCTTTAGACACCACGCAACCACC	
ZP1-sa-OT7-15-RP		TCCTCTACTCCAAAGGGTTTGTCATCAG
ZP1-sa-OT7-16-FP	TCATGAGCTAGACACCACGCAACCACC	
ZP1-sa-OT7-16-RP		CCTGAGATTCCAAAGGGTTTGTCATCAG
ZP1-sa-OT7-17-FP	TAGCGAGTTAGACACCACGCAACCACC	
ZP1-sa-OT7-17-RP		GTAGCTCCTCCAAAGGGTTTGTCATCAG
ZP1-sa-OT7-18-FP	TACTACGCTAGACACCACGCAACCACC	
ZP1-sa-OT7-18-RP		AGGCTCCGTCCAAAGGGTTTGTCATCAG
ZP1-sa-OT7-19-FP	GCAGCGTATAGACACCACGCAACCACC	
ZP1-sa-OT7-19-RP		CTGCGCATTCCAAAGGGTTTGTCATCAG
ZP1-sa-OT7-20-FP	GAGCGCTATAGACACCACGCAACCACC	
ZP1-sa-OT7-20-RP		CGCTCAGTTCCAAAGGGTTTGTCATCAG
ZP1-sa-OT8-11-FP	TTCTGCCTTTCAATAACCATCAAAGTCCCT	
ZP1-sa-OT8-11-RP		GCTCAGGATTGTCATAGTCAATAGTAAGCAGTCT
ZP1-sa-OT8-12-FP	AGGAGTCCTTCAATAACCATCAAAGTCCCT	
ZP1-sa-OT8-12-RP		CATGCCTATTGTCATAGTCAATAGTAAGCAGTCT
ZP1-sa-OT8-13-FP	GTAGAGAGTTCAATAACCATCAAAGTCCCT	
ZP1-sa-OT8-13-RP		CCTCTCTGTTGTCATAGTCAATAGTAAGCAGTCT
ZP1-sa-OT8-14-FP	AGCGTAGCTTCAATAACCATCAAAGTCCCT	
ZP1-sa-OT8-14-RP		CAGCCTCGTTGTCATAGTCAATAGTAAGCAGTCT
ZP1-sa-OT8-15-FP	TGCCTCTTTTCAATAACCATCAAAGTCCCT	
ZP1-sa-OT8-15-RP		TCCTCTACTTGTCATAGTCAATAGTAAGCAGTCT
ZP1-sa-OT8-16-FP	TCATGAGCTTCAATAACCATCAAAGTCCCT	
ZP1-sa-OT8-16-RP		CCTGAGATTTGTCATAGTCAATAGTAAGCAGTCT
ZP1-sa-OT8-17-FP	TAGCGAGTTTCAATAACCATCAAAGTCCCT	
ZP1-sa-OT8-17-RP		GTAGCTCCTTGTCATAGTCAATAGTAAGCAGTCT
ZP1-sa-OT8-18-FP	TACTACGCTTCAATAACCATCAAAGTCCCT	
ZP1-sa-OT8-18-RP		AGGCTCCGTTGTCATAGTCAATAGTAAGCAGTCT
ZP1-sa-OT8-19-FP	GCAGCGTATTCAATAACCATCAAAGTCCCT	
ZP1-sa-OT8-19-RP		CTGCGCATTTGTCATAGTCAATAGTAAGCAGTCT
ZP1-sa-OT8-20-FP	GAGCGCTATTCAATAACCATCAAAGTCCCT	
ZP1-sa-OT8-20-RP		CGCTCAGTTTGTCATAGTCAATAGTAAGCAGTCT
ZP1-sa-OT9-11-FP	TTCTGCCTTCTCAACCAAGTCAGGGAAATG	
ZP1-sa-OT9-11-RP		GCTCAGGAAAATCAGCACCTGCCTCCAC
ZP1-sa-OT9-12-FP	AGGAGTCCTCTCAACCAAGTCAGGGAAATG	
ZP1-sa-OT9-12-RP		CATGCCTAAAATCAGCACCTGCCTCCAC
ZP1-sa-OT9-13-FP	GTAGAGAGTCTCAACCAAGTCAGGGAAATG	
ZP1-sa-OT9-13-RP		CCTCTCTGAAATCAGCACCTGCCTCCAC
ZP1-sa-OT9-14-FP	AGCGTAGCTCTCAACCAAGTCAGGGAAATG	
ZP1-sa-OT9-14-RP		CAGCCTCGAAATCAGCACCTGCCTCCAC
ZP1-sa-OT9-15-FP	TGCCTCTTTCTCAACCAAGTCAGGGAAATG	

ZP1-sa-OT9-15-RP		TCCTCTACAAATCAGCACCTGCCTCCAC
ZP1-sa-OT9-16-FP	TCATGAGCTCTCAACCAAGTCAGGGAAATG	
ZP1-sa-OT9-16-RP		CCTGAGATAAATCAGCACCTGCCTCCAC
ZP1-sa-OT9-17-FP	TAGCGAGTTCTCAACCAAGTCAGGGAAATG	
ZP1-sa-OT9-17-RP		GTAGCTCCAAATCAGCACCTGCCTCCAC
ZP1-sa-OT9-18-FP	TACTACGCTCTCAACCAAGTCAGGGAAATG	
ZP1-sa-OT9-18-RP		AGGCTCCGAAATCAGCACCTGCCTCCAC
ZP1-sa-OT9-19-FP	GCAGCGTATCTCAACCAAGTCAGGGAAATG	
ZP1-sa-OT9-19-RP		CTGCGCATAAATCAGCACCTGCCTCCAC
ZP1-sa-OT9-20-FP	GAGCGCTATCTCAACCAAGTCAGGGAAATG	
ZP1-sa-OT9-20-RP		CGCTCAGTAAATCAGCACCTGCCTCCAC
ZP1-sa-OT10-11-FP	TTCTGCCTTCCTCTTGGCTGCTGAGACC	
ZP1-sa-OT10-11-RP		GCTCAGGAGAACCATTCCCATGAAGCTTTAC
ZP1-sa-OT10-12-FP	AGGAGTCCTCCTCTTGGCTGCTGAGACC	
ZP1-sa-OT10-12-RP		CATGCCTAGAACCATTCCCATGAAGCTTTAC
ZP1-sa-OT10-13-FP	GTAGAGAGTCCTCTTGGCTGCTGAGACC	
ZP1-sa-OT10-13-RP		CCTCTCTGGAACCATTCCCATGAAGCTTTAC
ZP1-sa-OT10-14-FP	AGCGTAGCTCCTCTTGGCTGCTGAGACC	
ZP1-sa-OT10-14-RP		CAGCCTCGGAACCATTCCCATGAAGCTTTAC
ZP1-sa-OT10-15-FP	TGCCTCTTTCCTCTTGGCTGCTGAGACC	
ZP1-sa-OT10-15-RP		TCCTCTACGAACCATTCCCATGAAGCTTTAC
ZP1-sa-OT10-16-FP	TCATGAGCTCCTCTTGGCTGCTGAGACC	
ZP1-sa-OT10-16-RP		CCTGAGATGAACCATTCCCATGAAGCTTTAC
ZP1-sa-OT10-17-FP	TAGCGAGTTCCTCTTGGCTGCTGAGACC	
ZP1-sa-OT10-17-RP		GTAGCTCCGAACCATTCCCATGAAGCTTTAC
ZP1-sa-OT10-18-FP	TACTACGCTCCTCTTGGCTGCTGAGACC	
ZP1-sa-OT10-18-RP		AGGCTCCGGAACCATTCCCATGAAGCTTTAC
ZP1-sa-OT10-19-FP	GCAGCGTATCCTCTTGGCTGCTGAGACC	
ZP1-sa-OT10-19-RP		CTGCGCATGAACCATTCCCATGAAGCTTTAC
ZP1-sa-OT10-20-FP	GAGCGCTATCCTCTTGGCTGCTGAGACC	
ZP1-sa-OT10-20-RP		CGCTCAGTGAACCATTCCCATGAAGCTTTAC
SLX2-target-1-FP	TAGATCGCGACTTTTGGCAACTTAAGGTTT	
SLX2-target-1-RP		CTCTCTATACCCCAACAGAGGAAGAACA
SLX2-target-2-FP	TATCCTCTGACTTTTGGCAACTTAAGGTTT	
SLX2-target-2-RP		AGAGTAGAACCCCAACAGAGGAAGAACA
SLX2-target-3-FP	GTAAGGAGGACTTTTGGCAACTTAAGGTTT	
SLX2-target-3-RP		ACTGCATAACCCCAACAGAGGAAGAACA
SLX2-target-4-FP	AAGGAGTAGACTTTTGGCAACTTAAGGTTT	
SLX2-target-4-RP		CTAAGCCTACCCCAACAGAGGAAGAACA
SLX2-target-5-FP	CGTCTAATGACTTTTGGCAACTTAAGGTTT	
SLX2-target-5-RP		TCTCTCCGACCCCAACAGAGGAAGAACA
SLX2-target-6-FP	TCGACTAGGACTTTTGGCAACTTAAGGTTT	
SLX2-target-6-RP		TTCTAGCTACCCCAACAGAGGAAGAACA
SLX2-target-7-FP	CCTAGAGTGACTTTTGGCAACTTAAGGTTT	
SLX2-target-7-RP		GCGTAAGAACCCCAACAGAGGAAGAACA
SLX2-target-8-FP	CTATTAAGGACTTTTGGCAACTTAAGGTTT	

SLX2-target-8-RP		AAGGCTATACCCCAACAGAGGAAGAACA
SLX2-target-9-FP	GAGCCTTAGACTTTTGGCAACTTAAGGTTT	
SLX2-target-9-RP		TTATGCGAACCCCAACAGAGGAAGAACA
SLX2-target-10-FP	TCGCCTTAGACTTTTGGCAACTTAAGGTTT	
SLX2-target-10-RP		CTAGTACGACCCCAACAGAGGAAGAACA

SLX2-sp-OT1-1-FP	TAGATCGCGGTTACAGCAAAGATGGCTG	
SLX2-sp-OT1-1-RP		CTCTCTATGTGACAAAGGGTGACTTAAGAGT
SLX2-sp-OT1-2-FP	TATCCTCTGGTTACAGCAAAGATGGCTG	
SLX2-sp-OT1-2-RP		AGAGTAGAGTGACAAAGGGTGACTTAAGAGT
SLX2-sp-OT1-3-FP	GTAAGGAGGGTTACAGCAAAGATGGCTG	
SLX2-sp-OT1-3-RP		ACTGCATAGTGACAAAGGGTGACTTAAGAGT
SLX2-sp-OT1-4-FP	AAGGAGTAGGTTACAGCAAAGATGGCTG	
SLX2-sp-OT1-4-RP		CTAAGCCTGTGACAAAGGGTGACTTAAGAGT
SLX2-sp-OT1-5-FP	CGTCTAATGGTTACAGCAAAGATGGCTG	
SLX2-sp-OT1-5-RP		TCTCTCCGGTGACAAAGGGTGACTTAAGAGT
SLX2-sp-OT1-6-FP	TCGACTAGGGTTACAGCAAAGATGGCTG	
SLX2-sp-OT1-6-RP		TTCTAGCTGTGACAAAGGGTGACTTAAGAGT
SLX2-sp-OT1-7-FP	CCTAGAGTGGTTACAGCAAAGATGGCTG	
SLX2-sp-OT1-7-RP		GCGTAAGAGTGACAAAGGGTGACTTAAGAGT
SLX2-sp-OT1-8-FP	CTATTAAGGGTTACAGCAAAGATGGCTG	
SLX2-sp-OT1-8-RP		AAGGCTATGTGACAAAGGGTGACTTAAGAGT
SLX2-sp-OT1-9-FP	GAGCCTTAGGTTACAGCAAAGATGGCTG	
SLX2-sp-OT1-9-RP		TTATGCGAGTGACAAAGGGTGACTTAAGAGT
SLX2-sp-OT1-10-FP	TCGCCTTAGGTTACAGCAAAGATGGCTG	
SLX2-sp-OT1-10-RP		CTAGTACGGTGACAAAGGGTGACTTAAGAGT
SLX2-sp-OT2-1-FP	TAGATCGCCACCATCTTCAACCAAACCT	
SLX2-sp-OT2-1-RP		CTCTCTATATTCAAGGAACAGGAAACAGA
SLX2-sp-OT2-2-FP	TATCCTCTCACCATCTTCAACCAAACCT	
SLX2-sp-OT2-2-RP		AGAGTAGAATTCAAGGAACAGGAAACAGA
SLX2-sp-OT2-3-FP	GTAAGGAGCACCATCTTCAACCAAACCT	
SLX2-sp-OT2-3-RP		ACTGCATAATTCAAGGAACAGGAAACAGA
SLX2-sp-OT2-4-FP	AAGGAGTACACCATCTTCAACCAAACCT	
SLX2-sp-OT2-4-RP		CTAAGCCTATTCAAGGAACAGGAAACAGA
SLX2-sp-OT2-5-FP	CGTCTAATCACCATCTTCAACCAAACCT	
SLX2-sp-OT2-5-RP		TCTCTCCGATTCAAGGAACAGGAAACAGA
SLX2-sp-OT2-6-FP	TCGACTAGCACCATCTTCAACCAAACCT	
SLX2-sp-OT2-6-RP		TTCTAGCTATTCAAGGAACAGGAAACAGA
SLX2-sp-OT2-7-FP	CCTAGAGTCACCATCTTCAACCAAACCT	
SLX2-sp-OT2-7-RP		GCGTAAGAATTCAAGGAACAGGAAACAGA
SLX2-sp-OT2-8-FP	CTATTAAGCACCATCTTCAACCAAACCT	
SLX2-sp-OT2-8-RP		AAGGCTATATTCAAGGAACAGGAAACAGA
SLX2-sp-OT2-9-FP	GAGCCTTACACCATCTTCAACCAAACCT	
SLX2-sp-OT2-9-RP		TTATGCGAATTCAAGGAACAGGAAACAGA
SLX2-sp-OT2-10-FP	TCGCCTTACACCATCTTCAACCAAACCT	
SLX2-sp-OT2-10-RP		CTAGTACGATTCAAGGAACAGGAAACAGA

SLX2-sp-OT3-1-FP	TAGATCGCAGATACCAACTACTCGGTTTCAG	

SLX2-sp-OT3-1-RP		CTCTCTATAGATTACTACTGCTGCATGTGAG
SLX2-sp-OT3-2-FP	TATCCTCTAGATACCAACTACTCGGTTTCAG	
SLX2-sp-OT3-2-RP		AGAGTAGAAGATTACTACTGCTGCATGTGAG
SLX2-sp-OT3-3-FP	GTAAGGAGAGATACCAACTACTCGGTTTCAG	
SLX2-sp-OT3-3-RP		ACTGCATAAGATTACTACTGCTGCATGTGAG
SLX2-sp-OT3-4-FP	AAGGAGTAAGATACCAACTACTCGGTTTCAG	
SLX2-sp-OT3-4-RP		CTAAGCCTAGATTACTACTGCTGCATGTGAG
SLX2-sp-OT3-5-FP	CGTCTAATAGATACCAACTACTCGGTTTCAG	
SLX2-sp-OT3-5-RP		TCTCTCCGAGATTACTACTGCTGCATGTGAG
SLX2-sp-OT3-6-FP	TCGACTAGAGATACCAACTACTCGGTTTCAG	
SLX2-sp-OT3-6-RP		TTCTAGCTAGATTACTACTGCTGCATGTGAG
SLX2-sp-OT3-7-FP	CCTAGAGTAGATACCAACTACTCGGTTTCAG	
SLX2-sp-OT3-7-RP		GCGTAAGAAGATTACTACTGCTGCATGTGAG
SLX2-sp-OT3-8-FP	CTATTAAGAGATACCAACTACTCGGTTTCAG	
SLX2-sp-OT3-8-RP		AAGGCTATAGATTACTACTGCTGCATGTGAG
SLX2-sp-OT3-9-FP	GAGCCTTAAGATACCAACTACTCGGTTTCAG	
SLX2-sp-OT3-9-RP		TTATGCGAAGATTACTACTGCTGCATGTGAG
SLX2-sp-OT3-10-FP	TCGCCTTAAGATACCAACTACTCGGTTTCAG	
SLX2-sp-OT3-10-RP		CTAGTACGAGATTACTACTGCTGCATGTGAG
SLX2-sp-OT4-1-FP	TAGATCGCGAAACTGTAAGCCAAACCCA	
SLX2-sp-OT4-1-RP		CTCTCTATTAATGGAGCCAAAGTAGGGG
SLX2-sp-OT4-2-FP	TATCCTCTGAAACTGTAAGCCAAACCCA	
SLX2-sp-OT4-2-RP		AGAGTAGATAATGGAGCCAAAGTAGGGG
SLX2-sp-OT4-3-FP	GTAAGGAGGAAACTGTAAGCCAAACCCA	
SLX2-sp-OT4-3-RP		ACTGCATATAATGGAGCCAAAGTAGGGG
SLX2-sp-OT4-4-FP	AAGGAGTAGAAACTGTAAGCCAAACCCA	
SLX2-sp-OT4-4-RP		CTAAGCCTTAATGGAGCCAAAGTAGGGG
SLX2-sp-OT4-5-FP	CGTCTAATGAAACTGTAAGCCAAACCCA	
SLX2-sp-OT4-5-RP		TCTCTCCGTAATGGAGCCAAAGTAGGGG
SLX2-sp-OT4-6-FP	TCGACTAGGAAACTGTAAGCCAAACCCA	
SLX2-sp-OT4-6-RP		TTCTAGCTTAATGGAGCCAAAGTAGGGG
SLX2-sp-OT4-7-FP	CCTAGAGTGAAACTGTAAGCCAAACCCA	
SLX2-sp-OT4-7-RP		GCGTAAGATAATGGAGCCAAAGTAGGGG
SLX2-sp-OT4-8-FP	CTATTAAGGAAACTGTAAGCCAAACCCA	
SLX2-sp-OT4-8-RP		AAGGCTATTAATGGAGCCAAAGTAGGGG
SLX2-sp-OT4-9-FP	GAGCCTTAGAAACTGTAAGCCAAACCCA	
SLX2-sp-OT4-9-RP		TTATGCGATAATGGAGCCAAAGTAGGGG
SLX2-sp-OT4-10-FP	TCGCCTTAGAAACTGTAAGCCAAACCCA	
SLX2-sp-OT4-10-RP		CTAGTACGTAATGGAGCCAAAGTAGGGG
SLX2-sp-OT5-1-FP	TAGATCGCCATCTTTCTGTAGGAATGTTGCT	
SLX2-sp-OT5-1-RP		CTCTCTATAAAACCTATGAGGTTCAGTAGCA
SLX2-sp-OT5-2-FP	TATCCTCTCATCTTTCTGTAGGAATGTTGCT	
SLX2-sp-OT5-2-RP		AGAGTAGAAAAACCTATGAGGTTCAGTAGCA
SLX2-sp-OT5-3-FP	GTAAGGAGCATCTTTCTGTAGGAATGTTGCT	
SLX2-sp-OT5-3-RP		ACTGCATAAAAACCTATGAGGTTCAGTAGCA
SLX2-sp-OT5-4-FP	AAGGAGTACATCTTTCTGTAGGAATGTTGCT	

SLX2-sp-OT5-4-RP CTAAGCCTAAAACCTATGAGGTTCAGTAGCA
SLX2-sp-OT5-5-FP CGTCTAATCATCTTTCTGTAGGAATGTTGCT
SLX2-sp-OT5-5-RP TCTCTCCGAAAACCTATGAGGTTCAGTAGCA
SLX2-sp-OT5-6-FP TCGACTAGCATCTTTCTGTAGGAATGTTGCT
SLX2-sp-OT5-6-RP TTCTAGCTAAAACCTATGAGGTTCAGTAGCA
SLX2-sp-OT5-7-FP CCTAGAGTCATCTTTCTGTAGGAATGTTGCT
SLX2-sp-OT5-7-RP GCGTAAGAAAAACCTATGAGGTTCAGTAGCA
SLX2-sp-OT5-8-FP CTATTAAGCATCTTTCTGTAGGAATGTTGCT
SLX2-sp-OT5-8-RP AAGGCTATAAAACCTATGAGGTTCAGTAGCA
SLX2-sp-OT5-9-FP GAGCCTTACATCTTTCTGTAGGAATGTTGCT
SLX2-sp-OT5-9-RP TTATGCGAAAAACCTATGAGGTTCAGTAGCA
SLX2-sp-OT5-10-FP TCGCCTTACATCTTTCTGTAGGAATGTTGCT
SLX2-sp-OT5-10-RP CTAGTACGAAAACCTATGAGGTTCAGTAGCA

SLX2-sp-OT6-1-FP TAGATCGCTGATTCAGTAAAGGTGGAGGA
SLX2-sp-OT6-1-RP CTCTCTATCAGGTGACAGATCGGAAGATA
SLX2-sp-OT6-2-FP TATCCTCTTGATTCAGTAAAGGTGGAGGA
SLX2-sp-OT6-2-RP AGAGTAGACAGGTGACAGATCGGAAGATA
SLX2-sp-OT6-3-FP GTAAGGAGTGATTCAGTAAAGGTGGAGGA
SLX2-sp-OT6-3-RP ACTGCATACAGGTGACAGATCGGAAGATA
SLX2-sp-OT6-4-FP AAGGAGTATGATTCAGTAAAGGTGGAGGA
SLX2-sp-OT6-4-RP CTAAGCCTCAGGTGACAGATCGGAAGATA
SLX2-sp-OT6-5-FP CGTCTAATTGATTCAGTAAAGGTGGAGGA
SLX2-sp-OT6-5-RP TCTCTCCGCAGGTGACAGATCGGAAGATA
SLX2-sp-OT6-6-FP TCGACTAGTGATTCAGTAAAGGTGGAGGA
SLX2-sp-OT6-6-RP TTCTAGCTCAGGTGACAGATCGGAAGATA
SLX2-sp-OT6-7-FP CCTAGAGTTGATTCAGTAAAGGTGGAGGA
SLX2-sp-OT6-7-RP GCGTAAGACAGGTGACAGATCGGAAGATA
SLX2-sp-OT6-8-FP CTATTAAGTGATTCAGTAAAGGTGGAGGA
SLX2-sp-OT6-8-RP AAGGCTATCAGGTGACAGATCGGAAGATA
SLX2-sp-OT6-9-FP GAGCCTTATGATTCAGTAAAGGTGGAGGA
SLX2-sp-OT6-9-RP TTATGCGACAGGTGACAGATCGGAAGATA
SLX2-sp-OT6-10-FP TCGCCTTATGATTCAGTAAAGGTGGAGGA
SLX2-sp-OT6-10-RP CTAGTACGCAGGTGACAGATCGGAAGATA

SLX2-sp-OT7-1-FP TAGATCGCGTTCGCCGTCCAGTAACC
SLX2-sp-OT7-1-RP CTCTCTATCAGACCATAGCTGTCCCGT
SLX2-sp-OT7-2-FP TATCCTCTGTTCGCCGTCCAGTAACC
SLX2-sp-OT7-2-RP AGAGTAGACAGACCATAGCTGTCCCGT
SLX2-sp-OT7-3-FP GTAAGGAGGTTCGCCGTCCAGTAACC
SLX2-sp-OT7-3-RP ACTGCATACAGACCATAGCTGTCCCGT
SLX2-sp-OT7-4-FP AAGGAGTAGTTCGCCGTCCAGTAACC
SLX2-sp-OT7-4-RP CTAAGCCTCAGACCATAGCTGTCCCGT
SLX2-sp-OT7-5-FP CGTCTAATGTTCGCCGTCCAGTAACC
SLX2-sp-OT7-5-RP TCTCTCCGCAGACCATAGCTGTCCCGT
SLX2-sp-OT7-6-FP TCGACTAGGTTCGCCGTCCAGTAACC
SLX2-sp-OT7-6-RP TTCTAGCTCAGACCATAGCTGTCCCGT
SLX2-sp-OT7-7-FP CCTAGAGTGTTCGCCGTCCAGTAACC

SLX2-sp-OT7-7-RP		GCGTAAGACAGACCATAGCTGTCCCGT
SLX2-sp-OT7-8-FP	CTATTAAGGTTCGCCGTCCAGTAACC	
SLX2-sp-OT7-8-RP		AAGGCTATCAGACCATAGCTGTCCCGT
SLX2-sp-OT7-9-FP	GAGCCTTAGTTCGCCGTCCAGTAACC	
SLX2-sp-OT7-9-RP		TTATGCGACAGACCATAGCTGTCCCGT
SLX2-sp-OT7-10-FP	TCGCCTTAGTTCGCCGTCCAGTAACC	
SLX2-sp-OT7-10-RP		CTAGTACGCAGACCATAGCTGTCCCGT
SLX2-sp-OT8-1-FP	TAGATCGCTCTCAGCCGTCATCGATAAT	
SLX2-sp-OT8-1-RP		CTCTCTATAAGGATGGGGGTGGTGTT
SLX2-sp-OT8-2-FP	TATCCTCTTCTCAGCCGTCATCGATAAT	
SLX2-sp-OT8-2-RP		AGAGTAGAAAGGATGGGGGTGGTGTT
SLX2-sp-OT8-3-FP	GTAAGGAGTCTCAGCCGTCATCGATAAT	
SLX2-sp-OT8-3-RP		ACTGCATAAAGGATGGGGGTGGTGTT
SLX2-sp-OT8-4-FP	AAGGAGTATCTCAGCCGTCATCGATAAT	
SLX2-sp-OT8-4-RP		CTAAGCCTAAGGATGGGGGTGGTGTT
SLX2-sp-OT8-5-FP	CGTCTAATTCTCAGCCGTCATCGATAAT	
SLX2-sp-OT8-5-RP		TCTCTCCGAAGGATGGGGGTGGTGTT
SLX2-sp-OT8-6-FP	TCGACTAGTCTCAGCCGTCATCGATAAT	
SLX2-sp-OT8-6-RP		TTCTAGCTAAGGATGGGGGTGGTGTT
SLX2-sp-OT8-7-FP	CCTAGAGTTCTCAGCCGTCATCGATAAT	
SLX2-sp-OT8-7-RP		GCGTAAGAAAGGATGGGGGTGGTGTT
SLX2-sp-OT8-8-FP	CTATTAAGTCTCAGCCGTCATCGATAAT	
SLX2-sp-OT8-8-RP		AAGGCTATAAGGATGGGGGTGGTGTT
SLX2-sp-OT8-9-FP	GAGCCTTATCTCAGCCGTCATCGATAAT	
SLX2-sp-OT8-9-RP		TTATGCGAAAGGATGGGGGTGGTGTT
SLX2-sp-OT8-10-FP	TCGCCTTATCTCAGCCGTCATCGATAAT	
SLX2-sp-OT8-10-RP		CTAGTACGAAGGATGGGGGTGGTGTT
SLX2-sp-OT9-1-FP	TAGATCGCAGAGCTGAAGTCACAGGCAG	
SLX2-sp-OT9-1-RP		CTCTCTATGAGGAAGATGGGGGAAATG
SLX2-sp-OT9-2-FP	TATCCTCTAGAGCTGAAGTCACAGGCAG	
SLX2-sp-OT9-2-RP		AGAGTAGAGAGGAAGATGGGGGAAATG
SLX2-sp-OT9-3-FP	GTAAGGAGAGAGCTGAAGTCACAGGCAG	
SLX2-sp-OT9-3-RP		ACTGCATAGAGGAAGATGGGGGAAATG
SLX2-sp-OT9-4-FP	AAGGAGTAAGAGCTGAAGTCACAGGCAG	
SLX2-sp-OT9-4-RP		CTAAGCCTGAGGAAGATGGGGGAAATG
SLX2-sp-OT9-5-FP	CGTCTAATAGAGCTGAAGTCACAGGCAG	
SLX2-sp-OT9-5-RP		TCTCTCCGGAGGAAGATGGGGGAAATG
SLX2-sp-OT9-6-FP	TCGACTAGAGAGCTGAAGTCACAGGCAG	
SLX2-sp-OT9-6-RP		TTCTAGCTGAGGAAGATGGGGGAAATG
SLX2-sp-OT9-7-FP	CCTAGAGTAGAGCTGAAGTCACAGGCAG	
SLX2-sp-OT9-7-RP		GCGTAAGAGAGGAAGATGGGGGAAATG
SLX2-sp-OT9-8-FP	CTATTAAGAGAGCTGAAGTCACAGGCAG	
SLX2-sp-OT9-8-RP		AAGGCTATGAGGAAGATGGGGGAAATG
SLX2-sp-OT9-9-FP	GAGCCTTAAGAGCTGAAGTCACAGGCAG	
SLX2-sp-OT9-9-RP		TTATGCGAGAGGAAGATGGGGGAAATG
SLX2-sp-OT9-10-FP	TCGCCTTAAGAGCTGAAGTCACAGGCAG	

SLX2-sp-OT9-10-RP		CTAGTACGGAGGAAGATGGGGGAAATG
SLX2-sp-OT10-1-FP	TAGATCGCACAGGAGATGGCAAGGCTA	
SLX2-sp-OT10-1-RP		CTCTCTATATGACACAGTGACGTGACATAAG
SLX2-sp-OT10-2-FP	TATCCTCTACAGGAGATGGCAAGGCTA	
SLX2-sp-OT10-2-RP		AGAGTAGAATGACACAGTGACGTGACATAAG
SLX2-sp-OT10-3-FP	GTAAGGAGACAGGAGATGGCAAGGCTA	
SLX2-sp-OT10-3-RP		ACTGCATAATGACACAGTGACGTGACATAAG
SLX2-sp-OT10-4-FP	AAGGAGTAACAGGAGATGGCAAGGCTA	
SLX2-sp-OT10-4-RP		CTAAGCCTATGACACAGTGACGTGACATAAG
SLX2-sp-OT10-5-FP	CGTCTAATACAGGAGATGGCAAGGCTA	
SLX2-sp-OT10-5-RP		TCTCTCCGATGACACAGTGACGTGACATAAG
SLX2-sp-OT10-6-FP	TCGACTAGACAGGAGATGGCAAGGCTA	
SLX2-sp-OT10-6-RP		TTCTAGCTATGACACAGTGACGTGACATAAG
SLX2-sp-OT10-7-FP	CCTAGAGTACAGGAGATGGCAAGGCTA	
SLX2-sp-OT10-7-RP		GCGTAAGAATGACACAGTGACGTGACATAAG
SLX2-sp-OT10-8-FP	CTATTAAGACAGGAGATGGCAAGGCTA	
SLX2-sp-OT10-8-RP		AAGGCTATATGACACAGTGACGTGACATAAG
SLX2-sp-OT10-9-FP	GAGCCTTAACAGGAGATGGCAAGGCTA	
SLX2-sp-OT10-9-RP		TTATGCGAATGACACAGTGACGTGACATAAG
SLX2-sp-OT10-10-FP	TCGCCTTAACAGGAGATGGCAAGGCTA	
SLX2-sp-OT10-10-RP		CTAGTACGATGACACAGTGACGTGACATAAG
SLX2-target-11-FP	TTCTGCCTGACTTTTGGCAACTTAAGGTTT	
SLX2-target-11-RP		GCTCAGGAACCCCAACAGAGGAAGAACA
SLX2-target-12-FP	AGGAGTCCGACTTTTGGCAACTTAAGGTTT	
SLX2-target-12-RP		CATGCCTAACCCCAACAGAGGAAGAACA
SLX2-target-13-FP	GTAGAGAGGACTTTTGGCAACTTAAGGTTT	
SLX2-target-13-RP		CCTCTCTGACCCCAACAGAGGAAGAACA
SLX2-target-14-FP	AGCGTAGCGACTTTTGGCAACTTAAGGTTT	
SLX2-target-14-RP		CAGCCTCGACCCCAACAGAGGAAGAACA
SLX2-target-15-FP	TGCCTCTTGACTTTTGGCAACTTAAGGTTT	
SLX2-target-15-RP		TCCTCTACACCCCAACAGAGGAAGAACA
SLX2-target-16-FP	TCATGAGCGACTTTTGGCAACTTAAGGTTT	
SLX2-target-16-RP		CCTGAGATACCCCAACAGAGGAAGAACA
SLX2-target-17-FP	TAGCGAGTGACTTTTGGCAACTTAAGGTTT	
SLX2-target-17-RP		GTAGCTCCACCCCAACAGAGGAAGAACA
SLX2-target-18-FP	TACTACGCGACTTTTGGCAACTTAAGGTTT	
SLX2-target-18-RP		AGGCTCCGACCCCAACAGAGGAAGAACA
SLX2-target-19-FP	GCAGCGTAGACTTTTGGCAACTTAAGGTTT	
SLX2-target-19-RP		CTGCGCATACCCCAACAGAGGAAGAACA
SLX2-target-20-FP	GAGCGCTAGACTTTTGGCAACTTAAGGTTT	
SLX2-target-20-RP		CGCTCAGTACCCCAACAGAGGAAGAACA
SLX2-sa-OT1-11-FP	TTCTGCCTGTGAAAGTAAATTAAATTTCCAAG	
SLX2-sa-OT1-11-RP		GCTCAGGACTAGACAGGAAATGGAAAAA
SLX2-sa-OT1-12-FP	AGGAGTCCGTGAAAGTAAATTAAATTTCCAAG	
SLX2-sa-OT1-12-RP		CATGCCTACTAGACAGGAAATGGAAAAA

SLX2-sa-OT1-13-FP	GTAGAGAGGTGAAAGTAAATTAAATTTCCAAG	
SLX2-sa-OT1-13-RP		CCTCTCTGCTAGACAGGAAATGGGAAAAA
SLX2-sa-OT1-14-FP	AGCGTAGCGTGAAAGTAAATTAAATTTCCAAG	
SLX2-sa-OT1-14-RP		CAGCCTCGCTAGACAGGAAATGGGAAAAA
SLX2-sa-OT1-15-FP	TGCCTCTTGTGAAAGTAAATTAAATTTCCAAG	
SLX2-sa-OT1-15-RP		TCCTCTACCTAGACAGGAAATGGGAAAAA
SLX2-sa-OT1-16-FP	TCATGAGCGTGAAAGTAAATTAAATTTCCAAG	
SLX2-sa-OT1-16-RP		CCTGAGATCTAGACAGGAAATGGGAAAAA
SLX2-sa-OT1-17-FP	TAGCGAGTGTGAAAGTAAATTAAATTTCCAAG	
SLX2-sa-OT1-17-RP		GTAGCTCCCTAGACAGGAAATGGGAAAAA
SLX2-sa-OT1-18-FP	TACTACGCGTGAAAGTAAATTAAATTTCCAAG	
SLX2-sa-OT1-18-RP		AGGCTCCGCTAGACAGGAAATGGGAAAAA
SLX2-sa-OT1-19-FP	GCAGCGTAGTGAAAGTAAATTAAATTTCCAAG	
SLX2-sa-OT1-19-RP		CTGCGCATCTAGACAGGAAATGGGAAAAA
SLX2-sa-OT1-20-FP	GAGCGCTAGTGAAAGTAAATTAAATTTCCAAG	
SLX2-sa-OT1-20-RP		CGCTCAGTCTAGACAGGAAATGGGAAAAA
SLX2-sa-OT2-11-FP	TTCTGCCTAAAGGAGCCAGAGTTCCAG	
SLX2-sa-OT2-11-RP		GCTCAGGATGTTCTTCACGTTTCTTGTCC
SLX2-sa-OT2-12-FP	AGGAGTCCAAAGGAGCCAGAGTTCCAG	
SLX2-sa-OT2-12-RP		CATGCCTATGTTCTTCACGTTTCTTGTCC
SLX2-sa-OT2-13-FP	GTAGAGAGAAAGGAGCCAGAGTTCCAG	
SLX2-sa-OT2-13-RP		CCTCTCTGTGTTCTTCACGTTTCTTGTCC
SLX2-sa-OT2-14-FP	AGCGTAGCAAAGGAGCCAGAGTTCCAG	
SLX2-sa-OT2-14-RP		CAGCCTCGTGTTCTTCACGTTTCTTGTCC
SLX2-sa-OT2-15-FP	TGCCTCTTAAAGGAGCCAGAGTTCCAG	
SLX2-sa-OT2-15-RP		TCCTCTACTGTTCTTCACGTTTCTTGTCC
SLX2-sa-OT2-16-FP	TCATGAGCAAAGGAGCCAGAGTTCCAG	
SLX2-sa-OT2-16-RP		CCTGAGATTGTTCTTCACGTTTCTTGTCC
SLX2-sa-OT2-17-FP	TAGCGAGTAAAGGAGCCAGAGTTCCAG	
SLX2-sa-OT2-17-RP		GTAGCTCCTGTTCTTCACGTTTCTTGTCC
SLX2-sa-OT2-18-FP	TACTACGCAAAGGAGCCAGAGTTCCAG	
SLX2-sa-OT2-18-RP		AGGCTCCGTGTTCTTCACGTTTCTTGTCC
SLX2-sa-OT2-19-FP	GCAGCGTAAAAGGAGCCAGAGTTCCAG	
SLX2-sa-OT2-19-RP		CTGCGCATTGTTCTTCACGTTTCTTGTCC
SLX2-sa-OT2-20-FP	GAGCGCTAAAAGGAGCCAGAGTTCCAG	
SLX2-sa-OT2-20-RP		CGCTCAGTTGTTCTTCACGTTTCTTGTCC
SLX2-sa-OT3-11-FP	TTCTGCCTCTTTCCCGTGTTGGTTGG	
SLX2-sa-OT3-11-RP		GCTCAGGAGTCCCAGGGTGAGGAAGA
SLX2-sa-OT3-12-FP	AGGAGTCCCTTTCCCGTGTTGGTTGG	
SLX2-sa-OT3-12-RP		CATGCCTAGTCCCAGGGTGAGGAAGA
SLX2-sa-OT3-13-FP	GTAGAGAGCTTTCCCGTGTTGGTTGG	
SLX2-sa-OT3-13-RP		CCTCTCTGGTCCCAGGGTGAGGAAGA
SLX2-sa-OT3-14-FP	AGCGTAGCCTTTCCCGTGTTGGTTGG	
SLX2-sa-OT3-14-RP		CAGCCTCGGTCCCAGGGTGAGGAAGA
SLX2-sa-OT3-15-FP	TGCCTCTTCTTTCCCGTGTTGGTTGG	
SLX2-sa-OT3-15-RP		TCCTCTACGTCCCAGGGTGAGGAAGA

SLX2-sa-OT3-16-FP	TCATGAGCCTTTCCCGTGTTGGTTGG	
SLX2-sa-OT3-16-RP		CCTGAGATGTCCCAGGGTGAGGAAGA
SLX2-sa-OT3-17-FP	TAGCGAGTCTTTCCCGTGTTGGTTGG	
SLX2-sa-OT3-17-RP		GTAGCTCCGTCCCAGGGTGAGGAAGA
SLX2-sa-OT3-18-FP	TACTACGCCTTTCCCGTGTTGGTTGG	
SLX2-sa-OT3-18-RP		AGGCTCCGGTCCCAGGGTGAGGAAGA
SLX2-sa-OT3-19-FP	GCAGCGTACTTTCCCGTGTTGGTTGG	
SLX2-sa-OT3-19-RP		CTGCGCATGTCCCAGGGTGAGGAAGA
SLX2-sa-OT3-20-FP	GAGCGCTACTTTCCCGTGTTGGTTGG	
SLX2-sa-OT3-20-RP		CGCTCAGTGTCCCAGGGTGAGGAAGA

SLX2-sa-OT4-11-FP	TTCTGCCTACATTTCTAGAGCACCGGAGT	
SLX2-sa-OT4-11-RP		GCTCAGGAACATCAAGTGAATCATAAAACATG
SLX2-sa-OT4-12-FP	AGGAGTCCACATTTCTAGAGCACCGGAGT	
SLX2-sa-OT4-12-RP		CATGCCTAACATCAAGTGAATCATAAAACATG
SLX2-sa-OT4-13-FP	GTAGAGAGACATTTCTAGAGCACCGGAGT	
SLX2-sa-OT4-13-RP		CCTCTCTGACATCAAGTGAATCATAAAACATG
SLX2-sa-OT4-14-FP	AGCGTAGCACATTTCTAGAGCACCGGAGT	
SLX2-sa-OT4-14-RP		CAGCCTCGACATCAAGTGAATCATAAAACATG
SLX2-sa-OT4-15-FP	TGCCTCTTACATTTCTAGAGCACCGGAGT	
SLX2-sa-OT4-15-RP		TCCTCTACACATCAAGTGAATCATAAAACATG
SLX2-sa-OT4-16-FP	TCATGAGCACATTTCTAGAGCACCGGAGT	
SLX2-sa-OT4-16-RP		CCTGAGATACATCAAGTGAATCATAAAACATG
SLX2-sa-OT4-17-FP	TAGCGAGTACATTTCTAGAGCACCGGAGT	
SLX2-sa-OT4-17-RP		GTAGCTCCACATCAAGTGAATCATAAAACATG
SLX2-sa-OT4-18-FP	TACTACGCACATTTCTAGAGCACCGGAGT	
SLX2-sa-OT4-18-RP		AGGCTCCGACATCAAGTGAATCATAAAACATG
SLX2-sa-OT4-19-FP	GCAGCGTAACATTTCTAGAGCACCGGAGT	
SLX2-sa-OT4-19-RP		CTGCGCATACATCAAGTGAATCATAAAACATG
SLX2-sa-OT4-20-FP	GAGCGCTAACATTTCTAGAGCACCGGAGT	
SLX2-sa-OT4-20-RP		CGCTCAGTACATCAAGTGAATCATAAAACATG

SLX2-sa-OT5-11-FP	TTCTGCCTAACTAACTTTCTAGACAAGGCTGG	
SLX2-sa-OT5-11-RP		GCTCAGGAAAAGGATTTTATTTTCTGTATTTCC
SLX2-sa-OT5-12-FP	AGGAGTCCAACTAACTTTCTAGACAAGGCTGG	
SLX2-sa-OT5-12-RP		CATGCCTAAAAGGATTTTATTTTCTGTATTTCC
SLX2-sa-OT5-13-FP	GTAGAGAGAACTAACTTTCTAGACAAGGCTGG	
SLX2-sa-OT5-13-RP		CCTCTCTGAAAGGATTTTATTTTCTGTATTTCC
SLX2-sa-OT5-14-FP	AGCGTAGCAACTAACTTTCTAGACAAGGCTGG	
SLX2-sa-OT5-14-RP		CAGCCTCGAAAGGATTTTATTTTCTGTATTTCC
SLX2-sa-OT5-15-FP	TGCCTCTTAACTAACTTTCTAGACAAGGCTGG	
SLX2-sa-OT5-15-RP		TCCTCTACAAAGGATTTTATTTTCTGTATTTCC
SLX2-sa-OT5-16-FP	TCATGAGCAACTAACTTTCTAGACAAGGCTGG	
SLX2-sa-OT5-16-RP		CCTGAGATAAAGGATTTTATTTTCTGTATTTCC
SLX2-sa-OT5-17-FP	TAGCGAGTAACTAACTTTCTAGACAAGGCTGG	
SLX2-sa-OT5-17-RP		GTAGCTCCAAAGGATTTTATTTTCTGTATTTCC
SLX2-sa-OT5-18-FP	TACTACGCAACTAACTTTCTAGACAAGGCTGG	
SLX2-sa-OT5-18-RP		AGGCTCCGAAAGGATTTTATTTTCTGTATTTCC

SLX2-sa-OT5-19-FP	GCAGCGTAAACTAACTTTCTAGACAAGGCTGG
SLX2-sa-OT5-19-RP	CTGCGCATAAAGGATTTTATTTTCTGTATTTCC
SLX2-sa-OT5-20-FP	GAGCGCTAAACTAACTTTCTAGACAAGGCTGG
SLX2-sa-OT5-20-RP	CGCTCAGTAAAGGATTTTATTTTCTGTATTTCC
SLX2-sa-OT6-11-FP	TTCTGCCTAACGAAAAAAGAGTCACAGGA
SLX2-sa-OT6-11-RP	GCTCAGGAAAAAGGATGGAGTTGTATGAGA
SLX2-sa-OT6-12-FP	AGGAGTCCAACGAAAAAAGAGTCACAGGA
SLX2-sa-OT6-12-RP	CATGCCTAAAAAGGATGGAGTTGTATGAGA
SLX2-sa-OT6-13-FP	GTAGAGAGAACGAAAAAAGAGTCACAGGA
SLX2-sa-OT6-13-RP	CCTCTCTGAAAAGGATGGAGTTGTATGAGA
SLX2-sa-OT6-14-FP	AGCGTAGCAACGAAAAAAGAGTCACAGGA
SLX2-sa-OT6-14-RP	CAGCCTCGAAAAGGATGGAGTTGTATGAGA
SLX2-sa-OT6-15-FP	TGCCTCTTAACGAAAAAAGAGTCACAGGA
SLX2-sa-OT6-15-RP	TCCTCTACAAAAGGATGGAGTTGTATGAGA
SLX2-sa-OT6-16-FP	TCATGAGCAACGAAAAAAGAGTCACAGGA
SLX2-sa-OT6-16-RP	CCTGAGATAAAAGGATGGAGTTGTATGAGA
SLX2-sa-OT6-17-FP	TAGCGAGTAACGAAAAAAGAGTCACAGGA
SLX2-sa-OT6-17-RP	GTAGCTCCAAAAGGATGGAGTTGTATGAGA
SLX2-sa-OT6-18-FP	TACTACGCAACGAAAAAAGAGTCACAGGA
SLX2-sa-OT6-18-RP	AGGCTCCGAAAAGGATGGAGTTGTATGAGA
SLX2-sa-OT6-19-FP	GCAGCGTAAACGAAAAAAGAGTCACAGGA
SLX2-sa-OT6-19-RP	CTGCGCATAAAAGGATGGAGTTGTATGAGA
SLX2-sa-OT6-20-FP	GAGCGCTAAACGAAAAAAGAGTCACAGGA
SLX2-sa-OT6-20-RP	CGCTCAGTAAAAGGATGGAGTTGTATGAGA
SLX2-sa-OT7-11-FP	TTCTGCCTCTAACCTTTTCCCCACTGTGT
SLX2-sa-OT7-11-RP	GCTCAGGATGATTGCTGGACTTCGATATT
SLX2-sa-OT7-12-FP	AGGAGTCCCTAACCTTTTCCCCACTGTGT
SLX2-sa-OT7-12-RP	CATGCCTATGATTGCTGGACTTCGATATT
SLX2-sa-OT7-13-FP	GTAGAGAGCTAACCTTTTCCCCACTGTGT
SLX2-sa-OT7-13-RP	CCTCTCTGTGATTGCTGGACTTCGATATT
SLX2-sa-OT7-14-FP	AGCGTAGCCTAACCTTTTCCCCACTGTGT
SLX2-sa-OT7-14-RP	CAGCCTCGTGATTGCTGGACTTCGATATT
SLX2-sa-OT7-15-FP	TGCCTCTTCTAACCTTTTCCCCACTGTGT
SLX2-sa-OT7-15-RP	TCCTCTACTGATTGCTGGACTTCGATATT
SLX2-sa-OT7-16-FP	TCATGAGCCTAACCTTTTCCCCACTGTGT
SLX2-sa-OT7-16-RP	CCTGAGATTGATTGCTGGACTTCGATATT
SLX2-sa-OT7-17-FP	TAGCGAGTCTAACCTTTTCCCCACTGTGT
SLX2-sa-OT7-17-RP	GTAGCTCCTGATTGCTGGACTTCGATATT
SLX2-sa-OT7-18-FP	TACTACGCCTAACCTTTTCCCCACTGTGT
SLX2-sa-OT7-18-RP	AGGCTCCGTGATTGCTGGACTTCGATATT
SLX2-sa-OT7-19-FP	GCAGCGTACTAACCTTTTCCCCACTGTGT
SLX2-sa-OT7-19-RP	CTGCGCATTGATTGCTGGACTTCGATATT
SLX2-sa-OT7-20-FP	GAGCGCTACTAACCTTTTCCCCACTGTGT
SLX2-sa-OT7-20-RP	CGCTCAGTTGATTGCTGGACTTCGATATT
SLX2-sa-OT8-11-FP	TTCTGCCTTTTTGTGTGTGTTGTGTACAGTG

SLX2-sa-OT8-11-RP		GCTCAGGAAATGTGTTGATTTTGTGAGGG
SLX2-sa-OT8-12-FP	AGGAGTCCTTTTGTGTGTGTTGTGTACAGTG	
SLX2-sa-OT8-12-RP		CATGCCTAAATGTGTTGATTTTGTGAGGG
SLX2-sa-OT8-13-FP	GTAGAGAGTTTTGTGTGTGTTGTGTACAGTG	
SLX2-sa-OT8-13-RP		CCTCTCTGAATGTGTTGATTTTGTGAGGG
SLX2-sa-OT8-14-FP	AGCGTAGCTTTTGTGTGTGTTGTGTACAGTG	
SLX2-sa-OT8-14-RP		CAGCCTCGAATGTGTTGATTTTGTGAGGG
SLX2-sa-OT8-15-FP	TGCCTCTTTTTTGTGTGTGTTGTGTACAGTG	
SLX2-sa-OT8-15-RP		TCCTCTACAATGTGTTGATTTTGTGAGGG
SLX2-sa-OT8-16-FP	TCATGAGCTTTTGTGTGTGTTGTGTACAGTG	
SLX2-sa-OT8-16-RP		CCTGAGATAATGTGTTGATTTTGTGAGGG
SLX2-sa-OT8-17-FP	TAGCGAGTTTTTGTGTGTGTTGTGTACAGTG	
SLX2-sa-OT8-17-RP		GTAGCTCCAATGTGTTGATTTTGTGAGGG
SLX2-sa-OT8-18-FP	TACTACGCTTTTGTGTGTGTTGTGTACAGTG	
SLX2-sa-OT8-18-RP		AGGCTCCGAATGTGTTGATTTTGTGAGGG
SLX2-sa-OT8-19-FP	GCAGCGTATTTTGTGTGTGTTGTGTACAGTG	
SLX2-sa-OT8-19-RP		CTGCGCATAATGTGTTGATTTTGTGAGGG
SLX2-sa-OT8-20-FP	GAGCGCTATTTTGTGTGTGTTGTGTACAGTG	
SLX2-sa-OT8-20-RP		CGCTCAGTAATGTGTTGATTTTGTGAGGG

SLX2-sa-OT9-11-FP	TTCTGCCTTGTCTGACACGTTGGGTTC	
SLX2-sa-OT9-11-RP		GCTCAGGAGCTGCTGGAGCCTTTCTAT
SLX2-sa-OT9-12-FP	AGGAGTCCTGTCTGACACGTTGGGTTC	
SLX2-sa-OT9-12-RP		CATGCCTAGCTGCTGGAGCCTTTCTAT
SLX2-sa-OT9-13-FP	GTAGAGAGTGTCTGACACGTTGGGTTC	
SLX2-sa-OT9-13-RP		CCTCTCTGGCTGCTGGAGCCTTTCTAT
SLX2-sa-OT9-14-FP	AGCGTAGCTGTCTGACACGTTGGGTTC	
SLX2-sa-OT9-14-RP		CAGCCTCGGCTGCTGGAGCCTTTCTAT
SLX2-sa-OT9-15-FP	TGCCTCTTTGTCTGACACGTTGGGTTC	
SLX2-sa-OT9-15-RP		TCCTCTACGCTGCTGGAGCCTTTCTAT
SLX2-sa-OT9-16-FP	TCATGAGCTGTCTGACACGTTGGGTTC	
SLX2-sa-OT9-16-RP		CCTGAGATGCTGCTGGAGCCTTTCTAT
SLX2-sa-OT9-17-FP	TAGCGAGTTGTCTGACACGTTGGGTTC	
SLX2-sa-OT9-17-RP		GTAGCTCCGCTGCTGGAGCCTTTCTAT
SLX2-sa-OT9-18-FP	TACTACGCTGTCTGACACGTTGGGTTC	
SLX2-sa-OT9-18-RP		AGGCTCCGGCTGCTGGAGCCTTTCTAT
SLX2-sa-OT9-19-FP	GCAGCGTATGTCTGACACGTTGGGTTC	
SLX2-sa-OT9-19-RP		CTGCGCATGCTGCTGGAGCCTTTCTAT
SLX2-sa-OT9-20-FP	GAGCGCTATGTCTGACACGTTGGGTTC	
SLX2-sa-OT9-20-RP		CGCTCAGTGCTGCTGGAGCCTTTCTAT

SLX2-sa-OT10-11-FP	TTCTGCCTACTGCTCCGGTCAGGAAC	
SLX2-sa-OT10-11-RP		GCTCAGGACAATCACAAACCTCACATATGG
SLX2-sa-OT10-12-FP	AGGAGTCCACTGCTCCGGTCAGGAAC	
SLX2-sa-OT10-12-RP		CATGCCTACAATCACAAACCTCACATATGG
SLX2-sa-OT10-13-FP	GTAGAGAGACTGCTCCGGTCAGGAAC	
SLX2-sa-OT10-13-RP		CCTCTCTGCAATCACAAACCTCACATATGG
SLX2-sa-OT10-14-FP	AGCGTAGCACTGCTCCGGTCAGGAAC	

SLX2-sa-OT10-14-RP	CAGCCTCGCAATCACAAACCTCACATATGG
SLX2-sa-OT10-15-FP	TGCCTCTTACTGCTCCGGTCAGGAAC
SLX2-sa-OT10-15-RP	TCCTCTACCAATCACAAACCTCACATATGG
SLX2-sa-OT10-16-FP	TCATGAGCACTGCTCCGGTCAGGAAC
SLX2-sa-OT10-16-RP	CCTGAGATCAATCACAAACCTCACATATGG
SLX2-sa-OT10-17-FP	TAGCGAGTACTGCTCCGGTCAGGAAC
SLX2-sa-OT10-17-RP	GTAGCTCCCAATCACAAACCTCACATATGG
SLX2-sa-OT10-18-FP	TACTACGCACTGCTCCGGTCAGGAAC
SLX2-sa-OT10-18-RP	AGGCTCCGCAATCACAAACCTCACATATGG
SLX2-sa-OT10-19-FP	GCAGCGTAACTGCTCCGGTCAGGAAC
SLX2-sa-OT10-19-RP	CTGCGCATCAATCACAAACCTCACATATGG
SLX2-sa-OT10-20-FP	GAGCGCTAACTGCTCCGGTCAGGAAC
SLX2-sa-OT10-20-RP	CGCTCAGTCAATCACAAACCTCACATATGG
